# Identification of SNP and SSR Markers in Finger Millet Using Next Generation Sequencing Technologies

**DOI:** 10.1371/journal.pone.0159437

**Published:** 2016-07-25

**Authors:** Davis Gimode, Damaris A. Odeny, Etienne P. de Villiers, Solomon Wanyonyi, Mathews M. Dida, Emmarold E. Mneney, Alice Muchugi, Jesse Machuka, Santie M. de Villiers

**Affiliations:** 1 Kenyatta University, P.O. Box 43844–00100, Nairobi, Kenya; 2 ICRISAT-Nairobi, P.O. Box 39063–00623, Nairobi, Kenya; 3 Center for Tropical Medicine, University of Oxford, Oxford OX3 7BN, United Kingdom; 4 University of Eldoret, P.O. Box 1125–30100, Eldoret, Kenya; 5 Maseno University, Private Bag, Maseno, Kenya; 6 Mikocheni Agricultural Research Institute, P.O. Box 6226, Dar-Es-Salaam, Tanzania; 7 ICRAF-Nairobi, P.O Box 30677, Nairobi, Kenya; 8 Pwani University, PO Box 195–80108, Kilifi, Kenya; National Institute of Plant Genome Research, INDIA

## Abstract

Finger millet is an important cereal crop in eastern Africa and southern India with excellent grain storage quality and unique ability to thrive in extreme environmental conditions. Since negligible attention has been paid to improving this crop to date, the current study used Next Generation Sequencing (NGS) technologies to develop both Simple Sequence Repeat (SSR) and Single Nucleotide Polymorphism (SNP) markers. Genomic DNA from cultivated finger millet genotypes KNE755 and KNE796 was sequenced using both Roche 454 and Illumina technologies. Non-organelle sequencing reads were assembled into 207 Mbp representing approximately 13% of the finger millet genome. We identified 10,327 SSRs and 23,285 non-homeologous SNPs and tested 101 of each for polymorphism across a diverse set of wild and cultivated finger millet germplasm. For the 49 polymorphic SSRs, the mean polymorphism information content (PIC) was 0.42, ranging from 0.16 to 0.77. We also validated 92 SNP markers, 80 of which were polymorphic with a mean PIC of 0.29 across 30 wild and 59 cultivated accessions. Seventy-six of the 80 SNPs were polymorphic across 30 wild germplasm with a mean PIC of 0.30 while only 22 of the SNP markers showed polymorphism among the 59 cultivated accessions with an average PIC value of 0.15. Genetic diversity analysis using the polymorphic SNP markers revealed two major clusters; one of wild and another of cultivated accessions. Detailed STRUCTURE analysis confirmed this grouping pattern and further revealed 2 sub-populations within wild *E*. *coracana* subsp. *africana*. Both STRUCTURE and genetic diversity analysis assisted with the correct identification of the new germplasm collections. These polymorphic SSR and SNP markers are a significant addition to the existing 82 published SSRs, especially with regard to the previously reported low polymorphism levels in finger millet. Our results also reveal an unexploited finger millet genetic resource that can be included in the regional breeding programs in order to efficiently optimize productivity.

## Introduction

Cultivated finger millet (*Eleusine coracana* subsp. *coracana*) is an annual grass that is widely cultivated as a staple food in eastern Africa and south Asia. It is tetraploid (2n = 4x = 36) and belongs to the Poaceae family and Chloridoideae sub-family [1]. Its origins can be traced to the highlands of Uganda and Ethiopia where it was likely domesticated around 5000 years ago from its wild progenitor *E*. *coracana* subsp. *africana*. Other wild species include *E*. *kigeziensis*, *E*. *floccifolia*, *E*. *intermedia*, *E*. *tristachya*, *E*. *jaegeri* and *E*. *indica*. Finger millet is a nutritious cereal high in protein, methionine and other essential amino acids. The small seeds can be stored for years without damage, making it an important food reserve in times of famine. The grain is used for bread, porridge, beer, soup and pudding. In countries where it is grown, it is commonly referred to as *Wimbi* (Swahili), *Bulo* (Uganda), *Tellebun* (Sudan) and *Ragi* (India) [2,3].

Despite finger millet’s importance as a subsistence crop, little attention has focused on improving production, probably because millets in general have been considered of little economic importance compared to maize, wheat and rice [4]. As a result, finger millet still lacks the required basic genomic resources for efficient breeding and remains one of the few cultivated cereal crops lacking a high-density genetic linkage map. There are currently <100 informative SSR markers available for finger millet [5,6,7], and only one study reported the identification of SNPs for this cereal using genotyping-by-sequencing (GBS) [8]. Nevertheless, the increasing affordability and access to Next Generation Sequencing (NGS) approaches facilitate the development of the genomic tools required to study complex traits and map quantitative trait loci (QTLs) of interest [9,10] in any crop, including finger millet.

Genetic markers have revolutionized crop improvement through the detection of DNA polymorphisms for precise, efficient and cost effective germplasm characterization and management. Such markers that have been used in finger millet include Random Amplified Polymorphic DNA (RAPD) [11,12], Inter Simple Sequence Repeats (ISSRs) [12], Random Fragment Length Polymorphisms (RFLPs), Amplified Fragment Length Polymorphisms (AFLPs), SSRs [5,6] and SNPs [8]. SSRs have become the markers of choice over the past decade for many crops including potato [13], rice [14] and wheat [15]. They remain the most commonly used marker for molecular analysis in finger millet and were among the markers used to construct the first genetic linkage map of finger millet [5]. More recently, Expressed Sequence Tags (EST)- derived SSRs have been developed for finger millet [16,17] although only a small percentage showed significant polymorphism across the accessions tested.

Cloning and hybridization-based SSR libraries and Sanger sequencing, which were used to develop the first 45 SSR markers for finger millet [5] can now be substituted with NGS, which generates larger numbers of sequences faster and cheaper. Roche 454 (Life Sciences) and Illumina platforms generate and process hundreds of thousands to millions of DNA templates in parallel resulting in low running costs per base of generated sequence and gigabase scale throughput [18], allowing the identification of large numbers of both SSR and SNP markers relatively cheaply. SSRs are PCR-based [19], highly polymorphic, hypervariable, co-dominant, reproducible, multi-allelic and distributed throughout the genome [20]. They can therefore be applied to finger millet improvement in genome-wide screens for variation and trait association, fingerprinting, genetic diversity analysis and genotyping [21,22].

On the other hand, SNPs have become the markers of choice for crop genotyping due to their abundance with up to 1 SNP per 140 bp being observed in rice [23]. They are co-dominant, bi-allelic, highly polymorphic, reproducible [24] and can be automated for high throughput genotyping. For these reasons, SNP markers are frequently used for genotyping large numbers of individuals for genomics-assisted breeding and genetic diversity applications. As an allotetraploid (AA and BB sub-genomes) with high levels of inbreeding, SNP discovery in finger millet can be challenging due to low polymorphism levels and high numbers of homeologous SNPs, which occur as a result of polymorphism between the AA and BB sub-genomes of the same individual. Using relevant filtering tools and stringent mapping parameters [25], SNP identification has been successful in several other polyploid crops including wheat [26], cotton [27], oats [28] and groundnut [29] and therefore can be successfully applied in finger millet. The current study capitalized on the power of NGS to develop additional SSRs as well as new SNP markers for finger millet using Roche 454 and Illumina sequencing.

## Materials and Methods

### Plant material

Finger millet genotypes KNE755 and KNE796 were used to generate sequence data for SSR and SNP marker development. Ten diverse finger millet genotypes (Table 1) that were used previously to assess polymorphism levels of published SSRs [5] were used to validate SSR markers in this study. Additional 89 genotypes (Table 1) were used to validate SNP markers. All the cultivated genotypes were obtained from the ICRISAT gene bank, the Tanzanian gene bank and the Gene Bank of Kenya. Maseno University, Kenya and Mikocheni Agricultural Research Institute (MARI), Tanzania kindly provided wild accessions. This study did not involve any endangered or protected species.

**Table 1:** Accessions of the genus *Eleusine* used for validating SSR and SNP markers.

Species	Accession name	Code^a^	Origin	Purpose in the study
***E*. *coracana* ssp. *coracana***	KNE796	31	Kenya	Generation of markers, SNP validation
	KNE755	56	Kenya	Generation of markers, SNP validation
	GBK-044047A	N/A	Kenya	SSR marker validation
	GBK-000414A	N/A	Kenya	SSR marker validation
	GBK-011135A	N/A	Kenya	SSR marker validation
	Sansamula	N/A	Tanzania	SSR marker validation
	Namakonta	N/A	Tanzania	SSR marker validation
	Ebega	N/A	Uganda	SSR marker validation
	Bulo	N/A	Uganda	SSR marker validation
	Emorumoru	N/A	Uganda	SSR marker validation
	IE2572	N/A	Minicore collection	SSR marker validation
	IE2957	N/A	Minicore collection	SSR marker validation
	GBK033383	32	Kenya	SNP marker validation
	GBK033384	33	Kenya	SNP marker validation
	GBK0333446	34	Kenya	SNP marker validation
	GBK0333407A	35	Kenya	SNP marker validation
	GBK0333408A	36	Kenya	SNP marker validation
	GBK0333445A	37	Kenya	SNP marker validation
	GBK0333449A	38	Kenya	SNP marker validation
	GBK0333452A	39	Kenya	SNP marker validation
	GBK0333454A	40	Kenya	SNP marker validation
	GBK0333455A	41	Kenya	SNP marker validation
	GBK0333456A	42	Kenya	SNP marker validation
	GBK0333457A	43	Kenya	SNP marker validation
	GBK0333458A	44	Kenya	SNP marker validation
	GBK0333459A	45	Kenya	SNP marker validation
	GBK0333460A	46	Kenya	SNP marker validation
	GBK033373A	47	Kenya	SNP marker validation
	GBK033376A	48	Kenya	SNP marker validation
	GBK033377A	49	Kenya	SNP marker validation
	GBK033378A	50	Kenya	SNP marker validation
	GBK033379A	51	Kenya	SNP marker validation
	GBK033380A	52	Kenya	SNP marker validation
	GBK033381A	53	Kenya	SNP marker validation
	GBK033382A	54	Kenya	SNP marker validation
	IMULA	55	Uganda	SNP marker validation
	P224	57	Uganda	SNP marker validation
	U15	81	Uganda	SNP marker validation
	TZA128	58	Tanzania	SNP marker validation
	TZA132	59	Tanzania	SNP marker validation
	TZA137	60	Tanzania	SNP marker validation
	TZA138	61	Tanzania	SNP marker validation
	TZA141	62	Tanzania	SNP marker validation
	TZA1628	63	Tanzania	SNP marker validation
	TZA1629	64	Tanzania	SNP marker validation
	TZA1632	65	Tanzania	SNP marker validation
	TZA1633	66	Tanzania	SNP marker validation
	TZA1634	67	Tanzania	SNP marker validation
	TZA1636	68	Tanzania	SNP marker validation
	TZA1637	69	Tanzania	SNP marker validation
	TZA1638	70	Tanzania	SNP marker validation
	TZA1640	71	Tanzania	SNP marker validation
	TZA1655	72	Tanzania	SNP marker validation
	TZA1656	73	Tanzania	SNP marker validation
	TZA1658	74	Tanzania	SNP marker validation
	TZA1659	75	Tanzania	SNP marker validation
	TZA1661	76	Tanzania	SNP marker validation
	TZA1662	77	Tanzania	SNP marker validation
	TZA1663	78	Tanzania	SNP marker validation
	TZA1665	79	Tanzania	SNP marker validation
	TZA1666	80	Tanzania	SNP marker validation
	MS19	82	Kenya	SNP marker validation
	MS17	83	Kenya	SNP marker validation
	LEN24	88	Ethiopia	SNP marker validation
	MS21	84	Kenya	SNP marker validation
	EDL34	85	Tanzania	SNP marker validation
	MS18	86	Kenya	SNP marker validation
	EDL25	89	Tanzania	SNP marker validation
	UG10	87	Uganda	SNP marker validation
***Wild accessions*, *true species not confirmed***	EDL30	1	Tanzania	SNP marker validation
	EDL15	2	Tanzania	SNP marker validation
	MS9	3	Kenya	SNP marker validation
	MS13	4	Kenya	SNP marker validation
	UG19	5	Uganda	SNP marker validation
	MS5	6	Kenya	SNP marker validation
	MS4	7	Kenya	SNP marker validation
	UG1	8	Uganda	SNP marker validation
	UG18	9	Uganda	SNP marker validation
	MSN10	10	Kenya	SNP marker validation
	MS8	11	Kenya	SNP marker validation
	AAU-ELU-48	12	Ethiopia	SNP marker validation
	UG9	13	Uganda	SNP marker validation
	UG11	14	Uganda	SNP marker validation
	UG20	15	Uganda	SNP marker validation
	MS3	16	Kenya	SNP marker validation
	MS6	17	Kenya	SNP marker validation
	MS7	18	Kenya	SNP marker validation
	MS11	19	Kenya	SNP marker validation
	MS12	20	Kenya	SNP marker validation
	MS15	21	Kenya	SNP marker validation
	EDL9	23	Tanzania	SNP marker validation
	EDL16	24	Tanzania	SNP marker validation
	LEN7	25	Ethiopia	SNP marker validation
	LESK10	26	Ethiopia	SNP marker validation
	MD48	22	Kenya	SNP marker validation
	UG3	27	Uganda	SNP marker validation
	UG8	28	Uganda	SNP marker validation
	MS16	29	Kenya	SNP marker validation
	EDL10	30	Tanzania	SNP marker validation

^a^This is the code used in STRUCTURE outputs. Genotypes that were not included in the STRUCTURE analysis are represented with N/A.

### Library preparation and sequencing

For Roche 454 sequencing, leaves of each genotype (KNE755 and KNE796) were sampled 2–3 weeks after planting, dried using silica gel and sent to Ecogenics (Schlieren, Switzerland) for DNA extraction, SSR enrichment and sequencing (Roche 454/FLX). For Illumina sequencing, DNA was extracted from two weeks old seedlings of KNE755 and KNE796 and sent to Georgia Genomics Facility at the University of Georgia (USA). A 1-μg portion of each DNA sample was fragmented using Covaris (Covaris Inc., MA, USA) ultrasonication. A second DNA portion of 5μg of each sample was digested using *PstI* methylation sensitive restriction endonuclease for 1 hour at 37°C in order to enrich for genic regions. After end-repair of both Covaris-sheared and enzyme digested DNA, sequencing libraries were prepared following the TruSeq protocol (Illumina, San Diego, USA) and sequenced on an Illumina Hi-Seq 2000.

### Processing of Illumina reads for SNP and SSR marker identification

Fastq-mcf [30] was used to remove adaptors and trim for quality. Finger millet chloroplast and mitochondrial sequences were removed by mapping trimmed reads to the rice reference chloroplast and mitochondrial genomes downloaded from GOBASE [31] using Bowtie2 [32]. *De novo* assembly of all non-organelle sequences was done using Velvet software [33] to create a reference file. Only reference contigs with at least 200 bp were maintained for marker identification and functional analysis. SSR motifs with a maximum of 4-nucleotide repeats were identified from the reference file using the software GMATo [34] with a minimum repeat value of 5. We specifically searched for only di-, tri-, and tetra-nucleotide repeats due to their abundance in plant genomes [35–37] and excluded other nucleotide repeats because of the high error rates [38] and less informative nature of mono-nucleotide repeats [39,40] and the low abundance of penta- and hexa-nucleotide repeats in monocots [41,42].

For SNP identification, BWA software [43] was used to map the Illumina reads from each genotype to the reference file. Generating reference sequences of each genotype and mapping back reads to the reference identified homeologous SNPs and their frequency in each genotype. SAMtools [44] was used to view and sort the mapped reads. Duplicate reads were removed from respective alignment sequences using Picard-tools 1.94 (http://picard.sourceforge.net) before running FreeBayes [45] to identify genetic variants. The raw SNPs obtained were filtered using VCFtools [46] based on a quality score of 30, maximum allele number of 2 and a minimum coverage of 3. Homeologous SNPs identified from each genotype were eliminated using VCFtools [46]. The raw data was submitted to the NCBI Sequence Read Archive (accession number SRP073162).

### Functional analysis

Reference sequence contigs that were at least 200bp long were masked for repetitive elements using RepeatMasker (http://www.repeatmasker.org/) and aligned using blastx [47] against rice genes retrieved from the UniProt database (http://www.uniprot.org/) as well as the non-redundant plant protein database retrieved from the Genbank (ftp://ftp.ncbi.nlm.nih.gov/refseq/release/plant/plant.1.protein.faa.gz) setting an e-value cutoff of 1e-5 and minimum similarity of 80%. A non-redundant protein list generated from the two databases was compiled and submitted to PANTHER [48] (http://pantherdb.org/) classification system for Gene Ontology (GO) term annotation (molecular function).

### SSR and SNP marker validation

Sequence assembly of Roche 454 data and identification of SSRs was done by Ecogenics (Switzerland). Primers were designed for 101 of the identified SSRs using the following parameters: primer length between 18–23 with an optimum of 21 bp, PCR products of 100 to 300 bp, primer TM between 58°- 64°C with an optimum of 60°C and GC content from 45–70%. All forward primers contained an M13-tag (5’- CACGACGTTGTAAAACGAC—3’) on the 5’ end that was fluorescently labelled to allow detection of amplification products [49]. SSR marker validation was performed across 10 selected genotypes as described by De Villiers et al. [7].

One hundred and one SNP markers were selected randomly and submitted for Competitive allele-specific PCR (KASPar) genotyping at LGC Genomics (http://www.lgcgenomics.com/genotyping/kasp_technical_resources/), UK. The data generated was viewed graphically as cluster plots using SNPviewer V2 (www.lgcgenomics.com). For SNP validation, two weeks-old seedlings of the 89 genotypes (Table 1) were sampled by placing 1 cm long leaf pieces in strip tubes supplied by LGC Genomics (www.lgcgroup.com). The tubes were sealed in a plastic bag with desiccant and immediately shipped to LGC Genomics (Herts, United Kingdom) for genotyping.

### Phylogenetic and population structure analysis

PowerMarker v.3.25 [50] was used to compute PIC and total numbers of alleles. Polymorphism information content (PIC) was calculated using the method of Botstein et al. [51] as below;

$$PIC=1-\overset{k}{\underset{i=0}{\sum }}p_{i}^{2}-\overset{k-1}{\underset{i=1}{\sum }}\overset{k}{\underset{j=i+1}{\sum }}2\;p_{i}^{2}p_{j}^{2}$$

Where, *p*_*i*_ and *p*_*j*_ are the frequencies of alleles *i* and *j*, respectively

The UPGMA based clustering was computed using TASSEL [52] and rooted using MD48, which belongs to the species *E*. *kigeziensis*. The genetic structure of finger millet accessions was determined using the admixture model with correlated allele frequencies based on the Monte Carlo Markov Chain (MCMC) algorithm implemented in STRUCTURE 2.3.3 software [53]. The admixture model assumed that the genome of each individual resulted from the mixture of *K* ancestral populations. The estimated proportions of each individual’s genotype originating from each of the *K* ancestral populations (q) was calculated for *K* ranging from 1 to 10 with 10 runs for each *K* value. For each run, a burn-in period of 10000 and MCMC replications of 100000 was used. The optimum *K* value was calculated using STRUCTURE HARVESTER [54], which computed the log likelihood of the data [LnP(D)] in the STRUCTURE output and an *ad hoc* statistic *Δk* based on the rate of change in LnP(D) between successive *k* [55]. Results from each replicate run were combined using the CLUMPP software [56].

## Results

### Sequence assembly

Table 2 provides a summary of reads generated and assembled for each genotype. Reads mapping to the rice organelle genomes were excluded from further analysis. The non-organelle finger millet reads from KNE755 (1,778,492) were assembled into 906,426 nodes/contigs consisting of 34,469,967 bp while those from KNE796 (5,706,821) were assembled into 5,552,610 nodes/contigs consisting of 167,333,449 bp (Table 2). All nuclear sequences from KNE755 and KNE796 were assembled into a reference fasta file containing 6,810,971 nodes/contigs spanning 207,197,804 bp. Assuming a genome size of 1.593 Gb [57], the assembled reads generated from both KNE755 and KNE796 genotypes represented about 13% of the finger millet genome. Contigs that were at least 200 bp long were retrieved from the reference file and used for SNP and SSR marker identification.

**Table 2:** A summary of sequencing reads generated for each genotype and the resulting assemblies.

Genotype	Roche 454 Raw Reads	Illumina Raw Reads	Nuclear Sequences (Reads)	Assembled Nuclear Sequences (bp)	Genome Coverage
**KNE755**	5,774	4,804,190	1,778,492	34,469,967	~2%
**KNE796**	5,266	13,007,430	5,706,821	167,333,449	~10%
**Combined Assembly**				207,197,804	~13%

### Homeologous SNPs

KNE755 was more abundant in homeologous SNPs with a frequency of 1/657 bp compared to a frequency of 1/956 bp in KNE796. The most abundant homeologous SNP in both genotypes was CT/AG (~62%) while CG was the rarest SNP at about 7.5%. The Ts/Tv ratios of the homeologous SNPs were comparable across the two genotypes but slightly higher in KNE755 (1.8) than in KNE796 (1.5).

### SSRs and Non-homeologous SNP mining

We identified 10,327 SSRs (di-, tri- and tetra-nucleotide repeats) (S1 Table) and 23,285 non-homeologous SNPs (S2 Table) from 77 Mbp compared to 38 SSRs and 1415 SNPs from the 1.3 Mbp putative genic regions. Table 3 shows a summary of SSRs and SNPs identified from different regions and the estimated frequencies of the markers across the two genotypes (KNE755 and KNE796). The most abundant SSRs were di-nucleotide repeats (80%), followed by tri-nucleotide (18.3%) and tetra-nucleotide repeats (1.2%) (Table 3). Of the di-nucleotide repeats, AG/CT was the most prevalent (39%) while CAA/TTG were the most prevalent (~9%) tri-nucleotide repeats. Within the putative genic regions, AT di-nucleotide repeats were the most abundant. The overall Ts/Tv SNP ratio was 1.8 compared to a ratio of 2 within the putative genic regions.

**Table 3:** A summary of identified SSR and SNP markers and their frequency across genotypes KNE796 and KNE755.

Marker Type	Type	Total Identified	Frequency
**SSR**	**ALL**	**10,327**	**1 per 7.5 Kb**
	di-nucleotide	8,308	1 per 9.3 kb
	Tri-nucleotide	1,895	1 per 40 kb
	Tetra-nucleotide	124	1 per 623 Kb
**Putative Genic SSRs**	**ALL**	**38**	**1 per 2 Mb**
	di-nucleotide SSR	27	1 per 2.9 Mb
	Tri-nucleotide	11	1 per 7 Mb
	Tetra-nucleotide	-	
**SNPs**	**ALL**	**23,285**	**1 per 3.3 kb**
	Transition	14962	1 per 5.1 Kb
	Transversion	8323	1 per 9.3 Kb
**Putative Genic SNPs**	**ALL**	**1,415**	**1 per 54.6 Kb**
	Transition	952	1 per 81 Kb
	Transversion	463	1 per 167 kb

### Functional annotation

**Fig 1:**
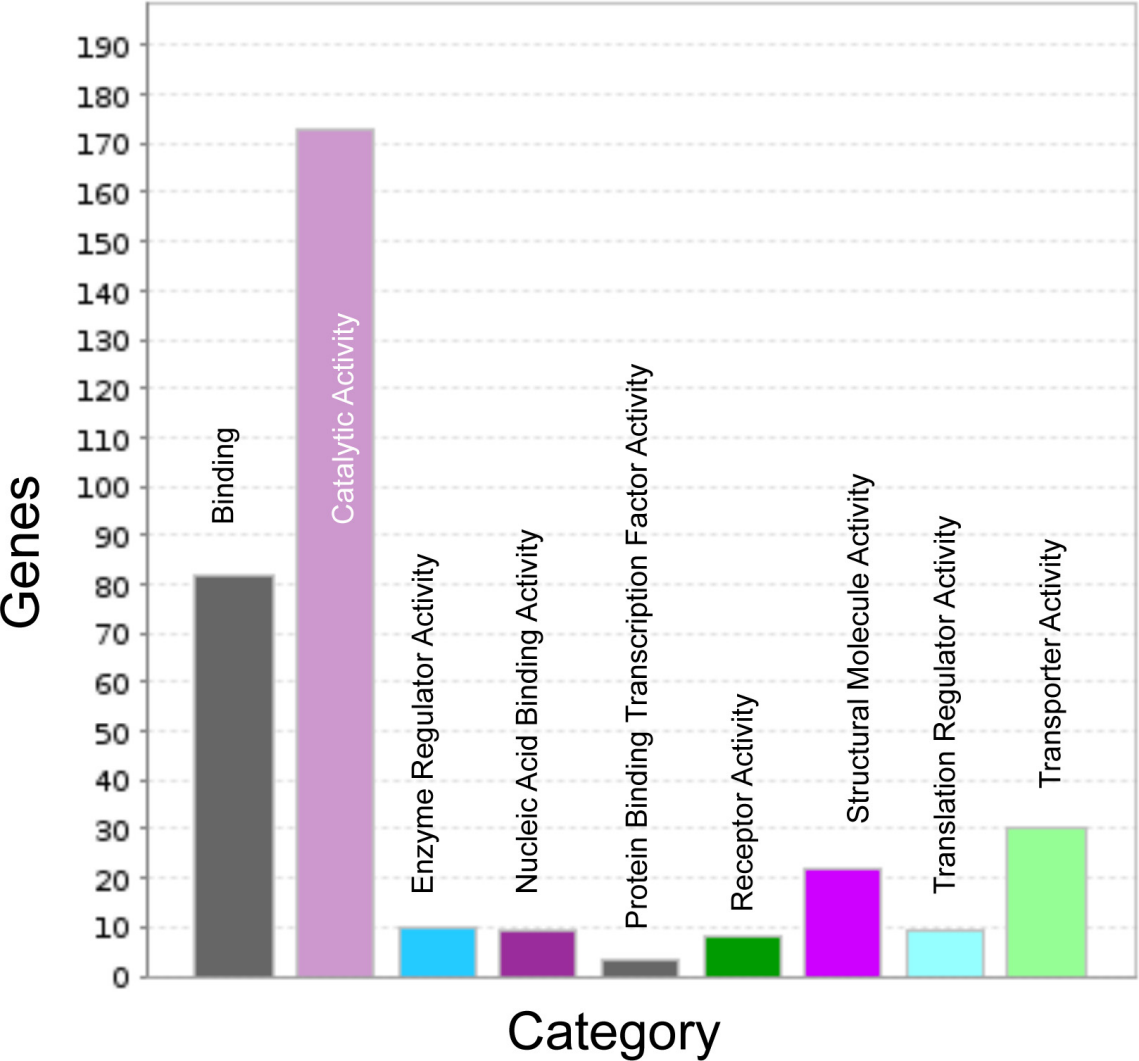
Molecular function categories. The distribution of SNP-containing putative genes that were assigned GO terms in PANTHER (http://pantherdb.org/). Catalytic activity category was over-represented.

Searches against the plant protein and rice gene databases revealed 6,821 (1,340,261 bp) and 6707 (1,328,367 bp) sequences respectively containing putative genes. While 9,175 and 11,632 of the reference contigs contained SSRs and SNPs respectively, only 36 and 564 of the putative genic sequences contained SNPs and SSRs respectively. Out of the 5,094 rice genes that showed sequence similarity with 6,707 finger millet sequences, 4,240 GO terms were retrieved for biological processes, 2,835 related to molecular functions and 1,147 to cellular components. For the 564 SNP-containing putative genic sequences, 519, 346 and 146 GO terms for biological processes, molecular functions and cellular components could be assigned respectively. Fig 1 shows the breakdown of different categories of the 346 GO terms for molecular functions as revealed by PANTHER. Genes with catalytic activity were over-represented (50%) followed by genes involved in binding (24%). Protein binding transcription factor activity was the least represented (1%).

### SSR and SNP validation using diverse finger millet genotypes

Of the 101 SSRs tested (S3 Table), 49 were polymorphic, 10 were monomorphic and 42 did not amplify products that could be scored unambiguously. Among the polymorphic markers, the PIC ranged from 0.16 to 0.77 with an average of 0.42 (Table 4). SSR loci ICECP54, ICECP47 and ICECP89 were the most polymorphic and revealed at least 5 alleles across the 10 genotypes. The rest of the markers revealed between 2–4 alleles with an average of 3 alleles per locus (Table 4).

**Table 4:** Characteristics of polymorphic SSRs after validation across 10 genotypes.

Marker	Major Allele Frequency	Allele Number	Gene Diversity	PIC
ICECP54	0.30	6	0.80	0.77
ICECP47	0.29	5	0.78	0.74
ICECP89	0.35	5	0.72	0.67
ICECP50	0.45	4	0.68	0.62
ICECP58	0.50	4	0.66	0.61
ICECP84	0.45	4	0.67	0.60
ICECP5	0.50	4	0.64	0.57
ICECP96	0.44	3	0.64	0.57
ICECP3	0.50	3	0.61	0.54
ICECP95	0.60	4	0.58	0.54
ICECP4	0.55	3	0.60	0.53
ICECP68	0.60	3	0.56	0.50
ICECP73	0.50	3	0.58	0.49
ICECP53	0.60	3	0.54	0.47
ICECP63	0.60	3	0.54	0.47
ICECP64	0.60	3	0.54	0.47
ICECP90	0.60	3	0.54	0.47
ICECP61	0.70	4	0.48	0.45
ICECP62	0.70	4	0.48	0.45
ICECP37	0.70	3	0.46	0.41
ICECP69	0.70	3	0.46	0.41
ICECP66	0.75	4	0.42	0.39
ICECP11	0.50	2	0.50	0.38
ICECP67	0.50	2	0.50	0.38
ICECP70	0.50	2	0.50	0.38
ICECP71	0.50	2	0.50	0.38
ICECP97	0.50	2	0.50	0.38
ICECP46	0.57	2	0.49	0.37
ICECP40	0.60	2	0.48	0.36
ICECP85	0.60	2	0.48	0.36
ICECP98	0.60	2	0.48	0.36
ICECP99	0.60	2	0.48	0.36
ICECP42	0.63	2	0.47	0.36
ICECP44	0.67	2	0.44	0.35
ICECP59	0.67	2	0.44	0.35
ICECP82	0.67	2	0.44	0.35
ICECP92	0.67	2	0.44	0.35
ICECP93	0.78	3	0.37	0.34
ICECP56	0.70	2	0.42	0.33
ICECP52	0.71	2	0.41	0.32
ICECP72	0.80	3	0.34	0.31
ICECP80	0.80	3	0.34	0.31
ICECP101	0.80	3	0.34	0.31
ICECP1	0.75	2	0.38	0.30
ICECP43	0.80	2	0.32	0.27
ICECP48	0.89	2	0.20	0.18
ICECP57	0.90	2	0.18	0.16
ICECP81	0.90	2	0.18	0.16
ICECP91	0.90	2	0.18	0.16
**Mean**	**0.62**	**3**	**0.48**	**0.42**

We developed 8,740 KASPar assays from 92 SNP regions across 93 finger millet accessions (S4 Table). The assays produced 8,099 identified allele calls, 640 unidentified allele calls and 1 bad call. The mean number of calls made per SNP was 87 with an allele call rate of 93%. Four genotypes that revealed > 80% missed calls and 12 SNP assays that revealed more than 90% unidentified allele calls as well as those that were monomorphic were excluded from further analysis. This resulted in 80 polymorphic markers (Table 5) tested across 89 genotypes (Table 1). The PIC ranged from 0.01 to 0.38 with a mean of 0.29 while heterozygosity ranged from 0 to 0.989 with a mean of 0.534 (Table 5). The most polymorphic markers were ICECSNT26 and ICECSNT94.

**Table 5:** A list of polymorphic SNP markers and their characteristics after validation across 89 *Eleusine* accessions.

SNP Locus	MAF	Gene Diversity	Heterozygosity	PIC (All)	PIC (Wild)	PIC (Cultivated)
**ICECSNT2**	0.70	0.42	0.057	0.33	0.18	N/A
**ICECSNT3**	0.98	0.03	0.034	0.03	0.09	N/A
**ICECSNT4**	0.76	0.37	0.077	0.30	0.23	0.02
**ICECSNT5**	0.54	0.50	0.857	0.37	0.37	0.37
**ICECSNT6**	0.52	0.50	0.966	0.37	0.37	N/A
**ICECSNT8**	0.51	0.50	0.955	0.37	0.37	0.37
**ICECSNT9**	0.52	0.50	0.966	0.37	0.37	N/A
**ICECSNT11**	0.52	0.50	0.966	0.37	0.37	N/A
**ICECSNT12**	0.85	0.25	0.092	0.22	0.37	0.03
**ICECSNT13**	0.92	0.15	0.045	0.14	0.27	N/A
**ICECSNT14**	0.52	0.50	0.966	0.37	0.37	0.03
**ICECSNT15**	0.97	0.07	0.000	0.06	0.16	N/A
**ICECSNT16**	0.52	0.50	0.966	0.37	0.37	N/A
**ICECSNT17**	0.51	0.50	0.977	0.37	0.37	N/A
**ICECSNT18**	0.70	0.42	0.081	0.33	0.22	N/A
**ICECSNT20**	0.51	0.50	0.943	0.37	0.37	N/A
**ICECSNT22**	0.75	0.38	0.082	0.31	0.29	N/A
**ICECSNT23**	0.51	0.50	0.977	0.37	0.37	N/A
**ICECSNT24**	0.74	0.38	0.060	0.31	0.21	N/A
**ICECSNT26**	0.50	0.50	0.977	0.38	0.38	N/A
**ICECSNT27**	0.52	0.50	0.954	0.37	0.37	0.37
**ICECSNT28**	0.51	0.50	0.966	0.37	0.37	N/A
**ICECSNT31**	0.52	0.50	0.966	0.37	0.37	N/A
**ICECSNT32**	0.67	0.44	0.038	0.34	0.19	0.16
**ICECSNT33**	0.99	0.01	0.011	0.01	N/A	0.02
**ICECSNT34**	0.51	0.50	0.989	0.37	0.37	N/A
**ICECSNT35**	0.58	0.49	0.000	0.37	0.18	0.27
**ICECSNT36**	0.69	0.43	0.047	0.34	0.22	0.12
**ICECSNT38**	0.96	0.08	0.011	0.07	0.17	0.02
**ICECSNT39**	0.70	0.42	0.045	0.33	0.20	N/A
**ICECSNT40**	0.99	0.02	0.000	0.02	0.06	N/A
**ICECSNT41**	0.52	0.50	0.966	0.37	0.37	N/A
**ICECSNT42**	0.62	0.47	0.713	0.36	0.17	N/A
**ICECSNT43**	0.52	0.50	0.964	0.37	0.37	N/A
**ICECSNT44**	0.52	0.50	0.966	0.37	0.37	N/A
**ICECSNT45**	0.61	0.48	0.778	0.36	0.19	N/A
**ICECSNT46**	0.99	0.01	0.012	0.01	0.03	N/A
**ICECSNT47**	0.52	0.50	0.966	0.37	0.37	N/A
**ICECSNT48**	0.52	0.50	0.913	0.37	0.37	0.37
**ICECSNT49**	0.88	0.21	0.171	0.19	0.36	0.02
**ICECSNT51**	0.61	0.47	0.722	0.36	0.19	N/A
**ICECSNT52**	0.89	0.20	0.150	0.18	0.37	N/A
**ICECSNT53**	0.51	0.50	0.977	0.37	0.37	N/A
**ICECSNT54**	0.97	0.07	0.000	0.06	0.16	N/A
**ICECSNT55**	0.74	0.39	0.482	0.31	0.38	0.21
**ICECSNT56**	0.54	0.50	0.871	0.37	0.36	N/A
**ICECSNT57**	0.51	0.50	0.966	0.37	0.37	N/A
**ICECSNT58**	0.51	0.50	0.951	0.37	0.38	0.37
**ICECSNT59**	0.96	0.07	0.000	0.07	0.17	N/A
**ICECSNT61**	0.63	0.47	0.744	0.36	0.08	N/A
**ICECSNT62**	0.70	0.42	0.000	0.33	0.12	N/A
**ICECSNT66**	0.82	0.29	0.000	0.25	0.33	N/A
**ICECSNT67**	0.97	0.07	0.000	0.06	0.17	N/A
**ICECSNT68**	0.52	0.50	0.965	0.37	0.37	N/A
**ICECSNT69**	0.98	0.05	0.000	0.04	0.12	N/A
**ICECSNT72**	0.97	0.07	0.023	0.06	N/A	0.09
**ICECSNT73**	0.52	0.50	0.966	0.37	0.37	N/A
**ICECSNT76**	0.52	0.50	0.966	0.37	0.37	N/A
**ICECSNT77**	0.72	0.40	0.069	0.32	0.24	N/A
**ICECSNT78**	0.99	0.01	0.011	0.01	N/A	0.02
**ICECSNT79**	0.94	0.11	0.000	0.11	0.26	N/A
**ICECSNT80**	0.51	0.50	0.989	0.37	0.37	N/A
**ICECSNT81**	0.52	0.50	0.966	0.37	0.37	N/A
**ICECSNT82**	0.52	0.50	0.965	0.37	0.37	N/A
**ICECSNT83**	0.69	0.43	0.198	0.34	0.30	0.16
**ICECSNT84**	0.52	0.50	0.966	0.37	0.37	N/A
**ICECSNT85**	0.67	0.44	0.074	0.35	0.20	0.11
**ICECSNT86**	0.85	0.25	0.115	0.22	0.37	N/A
**ICECSNT88**	0.55	0.49	0.894	0.37	0.34	N/A
**ICECSNT89**	0.51	0.50	0.977	0.37	0.37	N/A
**ICECSNT90**	0.75	0.38	0.061	0.30	0.22	N/A
**ICECSNT91**	0.52	0.50	0.966	0.37	0.37	N/A
**ICECSNT93**	0.72	0.40	0.000	0.32	0.29	0.15
**ICECSNT94**	0.50	0.50	0.977	0.38	0.38	N/A
**ICECSNT95**	0.80	0.32	0.267	0.27	0.37	0.03
**ICECSNT96**	0.80	0.32	0.179	0.27	0.35	N/A
**ICECSNT99**	0.52	0.50	0.966	0.37	0.37	N/A
**ICECSN99**	0.99	0.01	0.011	0.01	N/A	0.02
**ICECSN100**	0.52	0.50	0.966	0.37	0.37	N/A
**ICECSNT100**	0.52	0.50	0.966	0.37	0.37	N/A
**Mean**	0.67	0.38	0.524	0.29	0.30	0.15

MAF–Major Allele Frequency; PIC–Polymorphism Information Content

Markers that were monomorphic among wild and/or cultivated accessions are represented by “N/A” under the respective PIC columns

### Genetic diversity analysis

Ninety five % (76 out of 80) of the SNP markers showed polymorphism across 30 wild accessions while only 27.5% (22 out of 80) were polymorphic across the 59 cultivated genotypes revealing low variability within the cultivated finger millet. However, the 4 SNP markers (ICECSN99, ICECSNT33, ICECSNT72, ICECSNT78) that were monomorphic across wild accessions all showed polymorphism among the cultivated accessions. These polymorphisms resulted either from a single genotype harboring a heterozygous allele (for ICECSN99, ICECSNT33, and ICECSNT78) or from a few segregating genotypes (in the case of ICECSNT72). KNE796, which was also one of the genotypes used to generate the sequencing data, uniquely harbored the only heterozygous alleles for loci ICECSN99 and ICECSNT78 while P224, a popular high yielding variety, harbored the only heterozygous allele for locus ICECSNT33 and one of the 2 alternative homozygous alleles of locus ICECSNT72. The average PIC of the 80 SNP markers tested across 89 diverse genotypes was 0.29 (Table 5). The wild accessions revealed significantly higher polymorphism levels with 76 polymorphic markers and a mean PIC of 0.30. Only 22 SNP markers were polymorphic across the cultivated accessions, with an average PIC of 0.15 (Table 5).

**Fig 2:**
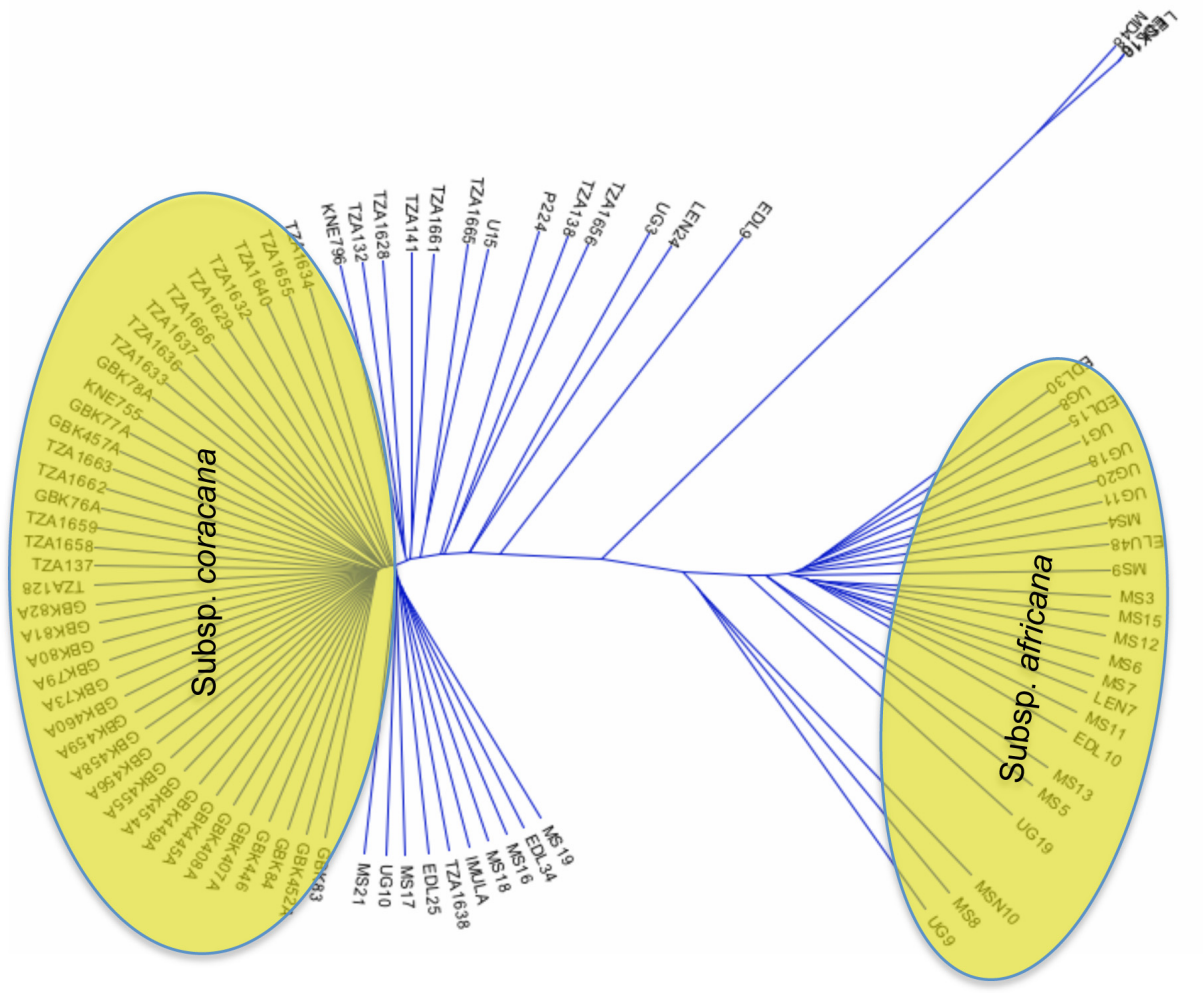
Clustering of 89 *Eleusine* spp using UPGMA. A dendrogram showing clustering of both wild and cultivated accessions generated using UPGMA in TASSEL software. Two major clusters (subsp. *coracana* and subsp. *africana*) are shown in yellow and one out-group consisting of 3 accessions (MD48, LESK10 and EDL16). Accessions MS19, MS17, MS21, EDL34, MS18, UG10, LEN24, EDL25, EDL9, MS16 and UG3, which were previously morphologically classified under subsp. *africana* clustered closer to the subsp. *coracana* genotypes and are therefore likely to be hybrids between subsp. *africana* and *coracana*.

The dendrogram (Fig 2) generated showed two major clusters (highlighted in yellow) and an outgroup formed by three *E*. *kigeziensis* accessions (MD48, LESK10 and EDL16). Cluster I comprised mainly of cultivated *E*. *coracana* subsp. *coracana* accessions while the second cluster comprised of *E*. *coracana* subsp. *africana* accessions (24 accessions). Accessions MS19, MS17, MS21, EDL34, MS18, UG10, LEN24, MS16 and EDL25, which were previously classified as wild, grouped closer to the subsp. *coracana* accessions, suggesting they may have hybridized with cultivated accessions.

**Fig 3:**
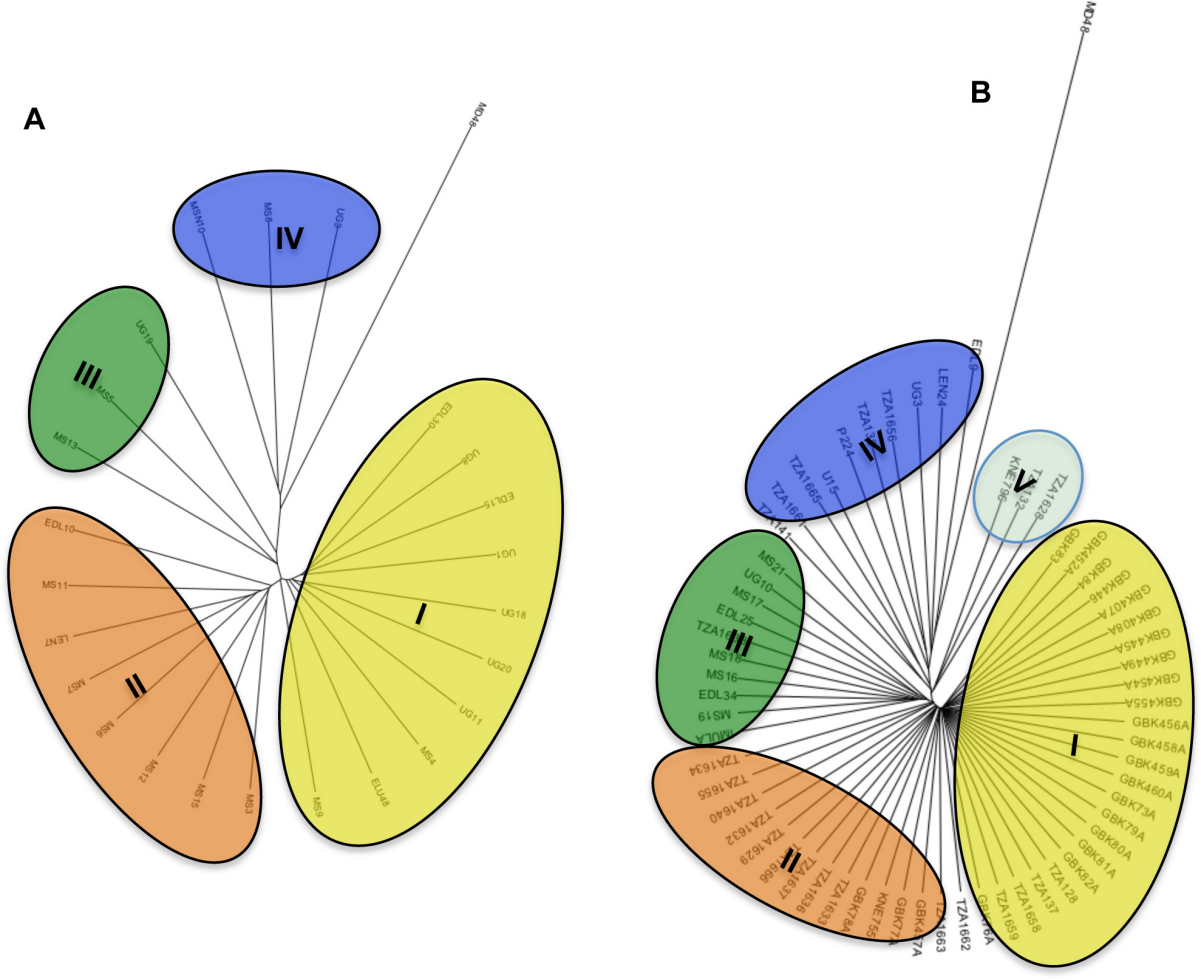
Comparison of clustering within the different sub species of *E*. *coracana*. Two dendrograms generated using genotypes that clustered under subsp. *africana* (A) and subsp. *coracana* cluster (B) in Fig 1 above, each rooted using MD48 (*E*. *kigeziensis*). In (A), 69 polymorphic markers were used to cluster 24 *africana* accessions while in (B), 71 polymorphic markers were used to cluster 62 accessions. Four major sub-clusters of *africana* are seen (I, II, III, IV), which correspond to similar sub-clusters in *coracana* (B). There is an additional cluster (V) in *coracana* (B), comprising only of high yielding improved accessions, which are likely to have been improved using Indian material.

We wanted to understand the extent of diversity within each of the 2 clusters identified in Fig 2 so we selected all accessions from each cluster and independently analysed them using *E*. *kigeziensis* accession MD48 as an outgroup. We generated two dendrograms (Fig 3), the first one composed of all the 24 accessions of *E*. *coracana* subsp. *africana* (Fig 3A) and the second one comprising of 62 accessions that had clustered within or closer to the *E*. *coracana* subsp. *coracana* group (Fig 3B). The 2 dendrograms showed relatively similar clustering patterns (clusters I, II, III, IV) except in Fig 3B, in which 3 genotypes (KNE796, TZA132 and TZA1628) formed an additional cluster V.

**Fig 4:**
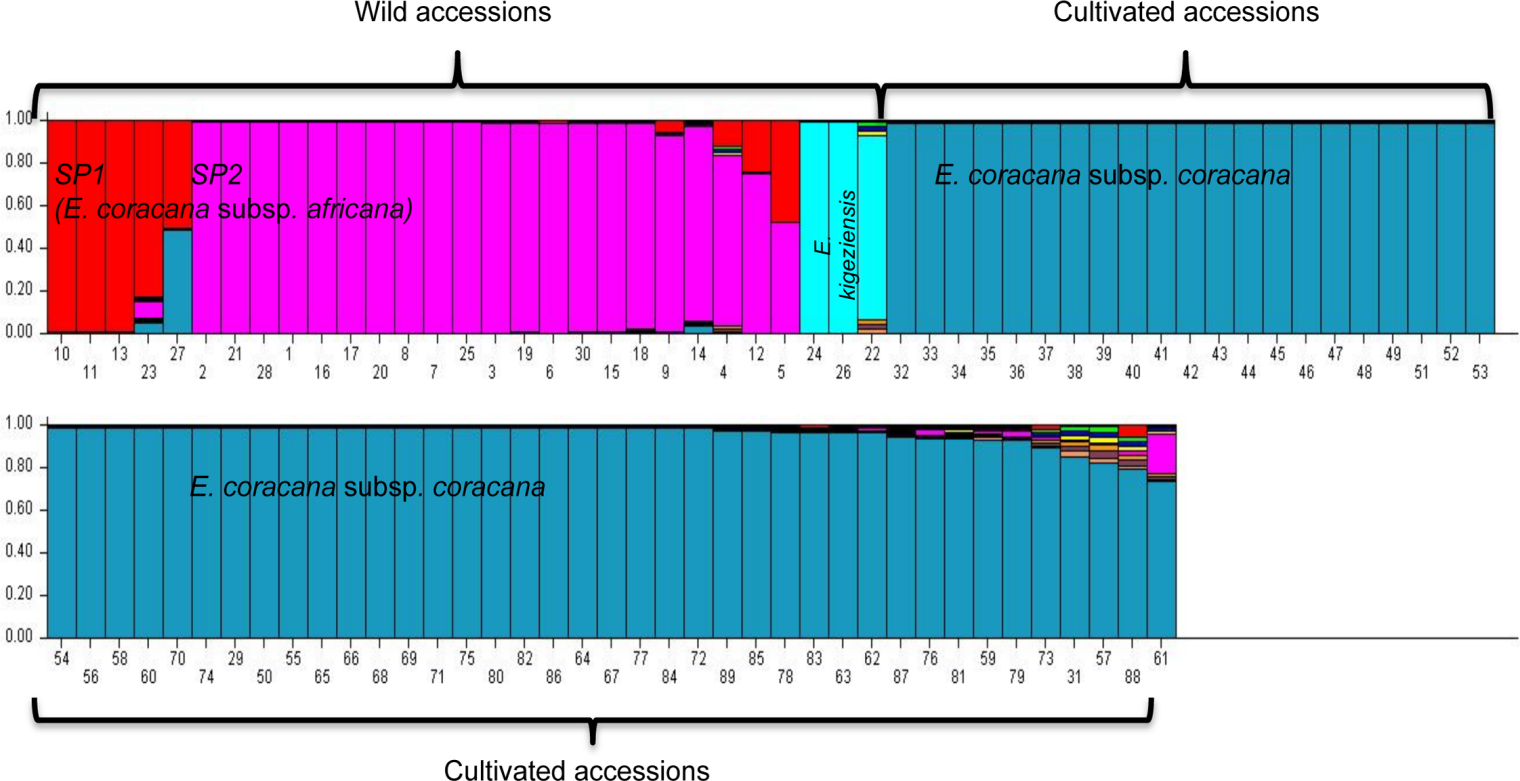
Population structure of 89 *Eleusine* accessions. Output of STRUCTURE analysis done using 80 polymorphic markers across 89 *Eleusine* accessions. Each vertical line represents an individual accession. The corresponding accession codes are provided in Table 1. The y-axis displays % estimated membership of each individual cluster or population. Accessions numbered 1 to 30 are assumed to be “wild”. The rest of the accessions are cultivated and were either collected from farmers’ fields or gene banks. Four sub-populations can be seen here highlighted in red (*E*. *coracana* subsp. *africana* sub-population 1), purple (*E*. *coracana* subsp. *africana* sub-population 2), turquoise (*E*. *kigeziensis)* and sky blue (*E*. *coracana* subsp. *coracana)*. Each color indicates the proportion of DNA segments for each individual, represented by a vertical bar, in each group.

The 89 accessions were further classified into two sub-populations (delta K = 2) (S1 Fig) using STRUCTURE. The sub-populations generated were complementary to the UPGMA tree analysis (Fig 2) and could distinguish the wild accessions (24 of subsp. *africana* and 3 of *E*. *kigeziensis*) from the cultivated (Fig 4). We further confirmed that the 9 accessions (MS19, MS21, MS18, MS17, EDL34, EDL25, LEN24, UG10, MS16) that had been previously recorded as subsp. *africana* were actually subsp. *coracana* as they grouped together with the cultivated accessions (Fig 4). Although LEN24 and UG10 showed some degree of admixture, there were also other cultivated genotypes such as TZA138, P224 and KNE796 that equally showed some levels of admixture but have been classified as *E*. *coracana* subsp *coracana*. The 3 out-group accessions (MD48, LESK10 and EDL16) revealed using UPGMA maintained a distinct grouping from the *E*. *coracana* subsp. *africana* accessions (Fig 4) confirming that they belonged to the distinct species *E*. *kigeziensis*.

**Fig 5:**
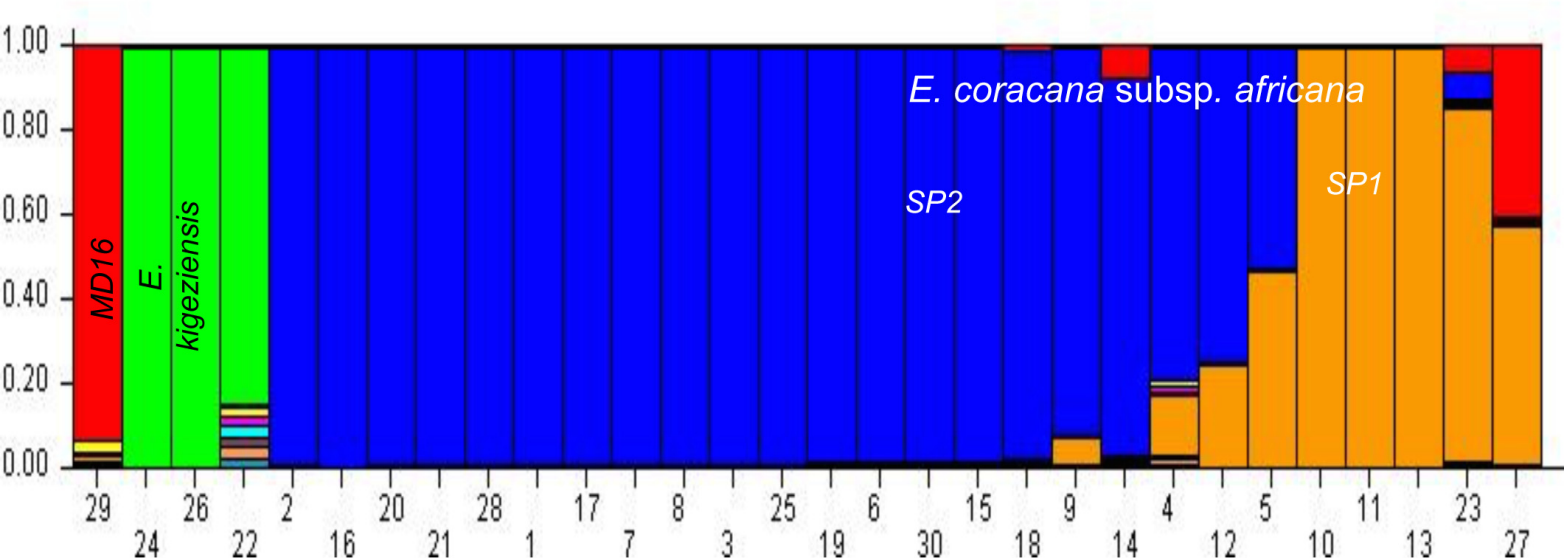
Population structure of 30 “wild” *Eleusine* accessions. Output of STRUCTURE analysis done using 76 polymorphic SNP markers across 30 “wild” accessions only. Four sub-populations are shown in red, green, blue and orange. The only genotype appearing under red (MS16) has been confirmed in Fig 2 to be cultivated. The 3 genotypes highlighted in green are *E*. *kigeziensis* (Lesk10, EDL16 and MD48) while blue and orange represent subsp. *africana* subpopulations 2 and 1 respectively. The orange sub-population is composed of the same samples clustering in red under Fig 3. The corresponding accession codes are shown in Table 1.

We subsequently explored the sub-populations within the wild accessions (three from *E*. *kigeziensis* and 24 of *E*. *coracana* subsp. *africana*) and attempted to correctly identify the two accessions (EDL9 and UG3) that were previously classified as wild but which now clustered with the cultivated accessions under both UPGMA clustering (Fig 2) and STRUCTURE (Fig 4) grouping. An independent STRUCTURE analysis of the 29 accessions, including MS16 as check, detected a maximum delta *K* at *K* = 3. The 30 “wild” accessions were classified into 3 sub-populations–one in *E*. *kigeziensis* and two in subpopulations of subsp. *africana*. Cultivated accession MS16 formed a distinct cluster (Fig 5). EDL9 and UG3 showed mixed ancestry with cultivated species further confirming their clustering with cultivated accessions in Fig 2. The latter STRUCTURE analysis also revealed minimal admixture of the two *E*. *kigeziensis* accessions (LESK10 and EDL16) with the other accessions in comparison with MD48 (Fig 5). This is understandable since MD48 has been used in the breeding program for a long time while LESK10 and EDL16 are new collections.

## Discussion

### Abundance of molecular markers in the finger millet genome

We report here the simultaneous mining of SSR and SNP markers from cultivated finger millet (*E*. *coracana* subsp. *coracana*), for which there are currently very few polymorphic markers. As expected, SNP markers were more abundant (1/3.3 kbp) than SSRs (1/7.5 kbp) despite the stringent filtering criteria used to eliminate homeologous SNPs. Homeologous SNPs were much more abundant (at least 1/kbp) suggesting a recent polyploidization event within the cultivated species. Recently formed polyploids have not undergone extensive genetic or genomic changes, and therefore their genomes would be additive with respect to their parental species [58]. The higher levels of homeologous SNPs may also suggest independent segregation of the *AA* and *BB* sub-genomes. The allotetraploid origin of finger millet remains unresolved and the detection of homeologous loci in the nuclear genome can provide critical information to elucidate its evolutionary history. Future evolution studies should include *E*. *indica* (AA), which is believed to be the maternal genome donor [59], alongside the two supspecies of *E*. *coracana*.

Consistent with previous studies in finger millet [6], there were fewer markers within the putative genic regions compared to those from genomic regions. The Ts/Tv ratio was higher within putative genic regions (2.0) compared with genomic regions (1.8). AT di-nucleotide repeats were the most abundant SSR markers within the genic regions. These results are in agreement with findings for *Petunia* [60] and mungbean [61] but lower than what was reported for rice [62]. Transitions (A/G, C/T) are always more common than Transversions (A/C, A/T, G/C, G/T) as they provide easy tolerance from selection pressure [63] but may also be an indication of low levels of genetic divergence [61].

We focused on di-, tri- and tetra-nucleotide SSRs due to their abundance in plant genomes [37–39] and selected those that were at least 10 bp long to maximize the polymorphism levels. Mononucleotide repeats were excluded due to the higher error rates [40] and less informative nature compared to di- and tri-nucleotide repeats [41,42]. Penta- and hexa-nucleotide repeats were similarly excluded due to low abundance as reported in monocot genomes [43,44]. The frequency of the chosen three classes of repeats (di-, tri-, tetra-) was 1/7.5 kb in finger millet genomic sequences. This was higher than observed in other grasses including foxtail millet (69/Mb) [64] and *Brachypodium* (101/Mb) [44] but lower than in rice [65] even though more classes of nucleotide repeats (di-, tri-, tetra-, penta-, hexa-) were considered in the latter study. In comparison with dicots, the SSR frequency distribution in finger millet was lower than in cucumber [66] and citrus [67]. However, meaningful SSR frequency distribution comparisons across species can only be done if SSR mining criteria and algorithms are fully standardized across studies [68].

The distribution of SNPs across the two genotypes (1/3.3 kb or 303 SNPs/Mb) was comparable to those observed between two *Japonica* rice varieties (346.6 SNPs/Mb) [69] but much lower than those reported across *Japonica* and *Indica* genomes [70]. However, the rice study identified SNPs from two *Japonica* varieties that included a landrace (*Omachi*) and an improved line (*Nipponbare*), whilst the current study included only improved finger millet varieties (KNE755 and KNE796), suggesting that higher numbers of SNP markers could be identified if more diverse genotypes were included in the discovery process. Stringent SNP identification criteria would need to be maintained to eliminate homeologous SNPs and reduce the numbers of false positives that have been observed in other polyploid crops [71]. Future SNP discovery studies in finger millet should also include genotypes from other *Eleusine* species, which are likely to be exploited in future breeding programs.

### Conversion of SNP markers into assays

KBiosciences allele-specific PCR (KASPar) technology was used to convert a random set of the identified SNPs into assays, as this system is flexible and was shown to work well with other polyploids [72,73]. Ninety-two of the 101 (91%) randomly selected SNP regions were successfully converted into assays, of which 80 (~80%) were polymorphic across across diverse *Eleusine* species. This conversion rate was quite compared to similar studies in hexaploid wheat (67%) [72] and polyploid *Spartina pectinata* (78.5%) [73], and demonstrated that KASPar-technology is suitable for quick validation of markers and low-throughput genotyping within and across different *Eleusine* species. However, for high-throughput SNP assays in the future, all available SNP genotyping platforms [74–76], as well as genotyping-by-sequencing (GBS) options [77,78] should be considered for the rapid analysis of high numbers of SNPs.

### SNP/SSR allelic diversity

In most plants, di-nucleotide SSRs have been reported to be the most polymorphic [79], followed by tri-nucleotide [37] repeats. The 12 most polymorphic SSR markers (PIC ≥ 0.5) in the current study were di-nucleotide repeats with a minimum of 22 bp in length. Previous studies on SSR marker development in finger millet reported the same [7]. Although most of the SSRs identified in this study were not further tested across diverse finger millet germplasm, we recommend that future investigations intending to make use of SSRs in finger millet should focus on testing mainly di-nucleotide repeats with a minimum of 20 bp in length.

The allelic diversity of the SNP markers identified in this study was relatively low (mean PIC of 0.29) and remarkably different across wild (mean PIC = 0.30) and cultivated (mean PIC = 0.15) germplasm. Narrow genetic diversity had been observed in cultivated finger millet [17,80] due to high inbreeding levels. Compared with wild species in which 76 SNP markers showed polymorphism, there were only 22 polymorphic markers across the cultivated species. Similar low PIC values were reported for other polyploids including wheat [81]. However, it must be noted that unlike SSRs, SNPs are bi-allelic with a maximum PIC values of 0.5 [81]. Since this was one of the few SNP diversity analysis studies in finger millet, additional studies are recommended before detailed conclusions can be made. Nevertheless, the abundant diversity observed among the wild accessions should be used in breeding programs to broaden the genetic base of cultivated accessions.

### Finger millet genetic diversity

The 80 polymorphic SNP markers developed in the current investigation successfully discriminated various *Eleusine* species and enabled correct classification of unknown genotypes. Most wild accessions used in the current study were new collections that were expected to be cross-compatible with cultivated species except MD48. The clustering pattern observed when all accessions were analysed together clearly distinguished between *E*. *coracana* subsp. *coracana* and that of subsp. *africana* confirming the distinctness of the two subspecies. We were also able to classify ten “unidentified” new collections (MS16, MS19, MS17, MS21, EDL34, MS18, UG10, LEN24, EDL25, EDL16, LESK10) correctly based on the phylogenetic and STRUCTURE analysis. Although MS16 was previously considered a wild accession belonging to *E*. *coracana* subsp. *africana*, our clustering confirmed it was a cultivated accession with mixed ancestry. Although Lesk10 and EDL16 were previously classified as *E*. *coracana* subsp. *africana*, their consistent clustering with MD48 (*E*. *kigeziensis*) left no doubt that these two genotypes belong to the same (*E*. *kigeziensis)* species. Due to the tight clustering between Lesk10 and EDL16, it was difficult to conclude whether the samples were contaminated or if the genotypes were indeed genetically very similar. More studies are needed to combine both morphological and genetic analysis to correctly distinguish these two genotypes (Lesk10 and EDL16) in the future.

Some degree of geographical clustering was also observed within the two major clusters. For example, within the *africana* subspecies, most of the accessions from Uganda (UG11, UG20, UG18, UG1) and western Kenya (at the border with Uganda) clustered together, while the Kenyan (names starting with GBK) and Tanzanian (names starting with TZA) cultivated accessions also clustered together. The deviation of some of the improved varieties from the major cluster may have been due to gene flow between subsp. *africana* and *coracana*, as well as the recent history of finger millet breeding in eastern Africa, which was influenced by Indian accessions. Some finger millet varieties released in Africa (such as P224) were bred through hybridization of African and Indian germplasm [18]. Previous studies including both African and Asian collections often resulted in distinct clustering of Asian accessions from African ones [17,82,83]. More surprising was the clustering of EDL9 and UG3 with the subsp. *coracana* accessions. Both EDL9 and UG3 were previously classified as belonging to subsp. *africana* using morphological features. Given the high levels of gene flow between *africana* and *coracana*, it is likely possible that these two genotypes have crossed with *coracana* and are therefore genetically more similar to subsp. *coracana* but morphologically similar to subsp. *africana*.

### Population structure

Eastern Africa, being the center of diversity for finger millet, contains a large number of landraces as well as finger millet wild relatives. Using STRUCTURE software, we identified four distinct sup-populations when *K* = 10 (Fig 4) representing *E*. *kigeziensis*, two sub-populations of *E*. *coracana* subsp. *africana* and *E*. *coracana* subsp. *coracana*. Of the three genotypes (MD48, LESK10 and EDL16) that clustered under *E*. *kigeziensis*, it was only MD48 that displayed some degree of admixture. MD48 was originally collected from Uganda [80], but had been used in the breeding program at Maseno University (Kenya) for many years. MD48 may have received pollen from other cross-compatible accessions growing side-by-side, although LESK10 and/or EDL16, appeared to be pure and could therefore be used in future studies aiming to capture pure *E*. *kigeziensis* genomes. While earlier studies within *E*. *coracana* species reported two subpopulations of *coracana* (Asian and African subpopulations) [80], this is the first study suggesting two subpopulations within subsp. *africana*. The two subpopulations of *africana* observed did not reveal any geographical clustering pattern and therefore need to be investigated further to determine the basis for grouping.

All *E*. *coracana* subsp. *coracana* accessions formed one distinct grouping and also enabled further identification of previously misidentified accessions, such as MS16. We were also able to confirm that both EDL9 and UG3 were admixtures and contained DNA from subsp. *coracana*. Clearly, UPGMA and morphological characterization alone are not enough for correct classification of accessions within a genus where gene flow is rampant. Other studies that included both Asian and African accessions in the past [80] have revealed two subpopulations of subsp. *coracana* that reflected the two geographies. Our study specifically included only eastern African collections of subsp *coracana*, and this may explain the low levels of genetic diversity and the observation of only one subpopulation of *coracana*.

This study illustrated the power of NGS to advance research in previously under-studied crops such as finger millet and demonstrated the immediate application of such resources in breeding programs. Although genic molecular markers are considered more useful due to their likely association with functional genes and wider application across related species, genomic markers are still extremely useful in finger millet due to their abundance and high polymorphism levels that would facilitate the immediate implementation of genomics-assisted breeding. No doubt, future marker development studies in finger millet would need to exploit the decreasing sequencing costs in order to generate higher numbers of genic markers as has been done in other polyploids [27,73]. While the narrow genetic diversity within cultivated finger millet can be immediately addressed through the exploitation of primary, secondary and tertiary genepools, such an effort will also require an extensive assembly of a well-characterized genetic resource alongside well-developed genomic resources. Finger millet breeders will therefore need to modernize their breeding tools in order to reduce the current tedious varietal development process, especially in Africa.

## Supporting Information

S1 Fig Optimum number of predicted sub-populations. (TIF)

S1 Table A summary of all SSRs identified and the corresponding contig name. (XLS)

S2 Table A summary of all SNPs identified and the corresponding contig name. (XLSX)

S3 Table A list of the 101 SSR primers supplied by Ecogenics and validated using 10 finger millet genotypes. (DOCX)

S4 Table A list of 92 SNP markers from which finger millet KASPar assays were developed. (DOCX)
