# Supplementary figures and images for: Identification of SNP and SSR Markers in Finger Millet Using Next Generation Sequencing Technologies
Source: PLoS One. 2016 Jul 25;11(7):e0159437. doi: 10.1371/journal.pone.0159437 (PMC4959724; doi:10.1371/journal.pone.0159437)

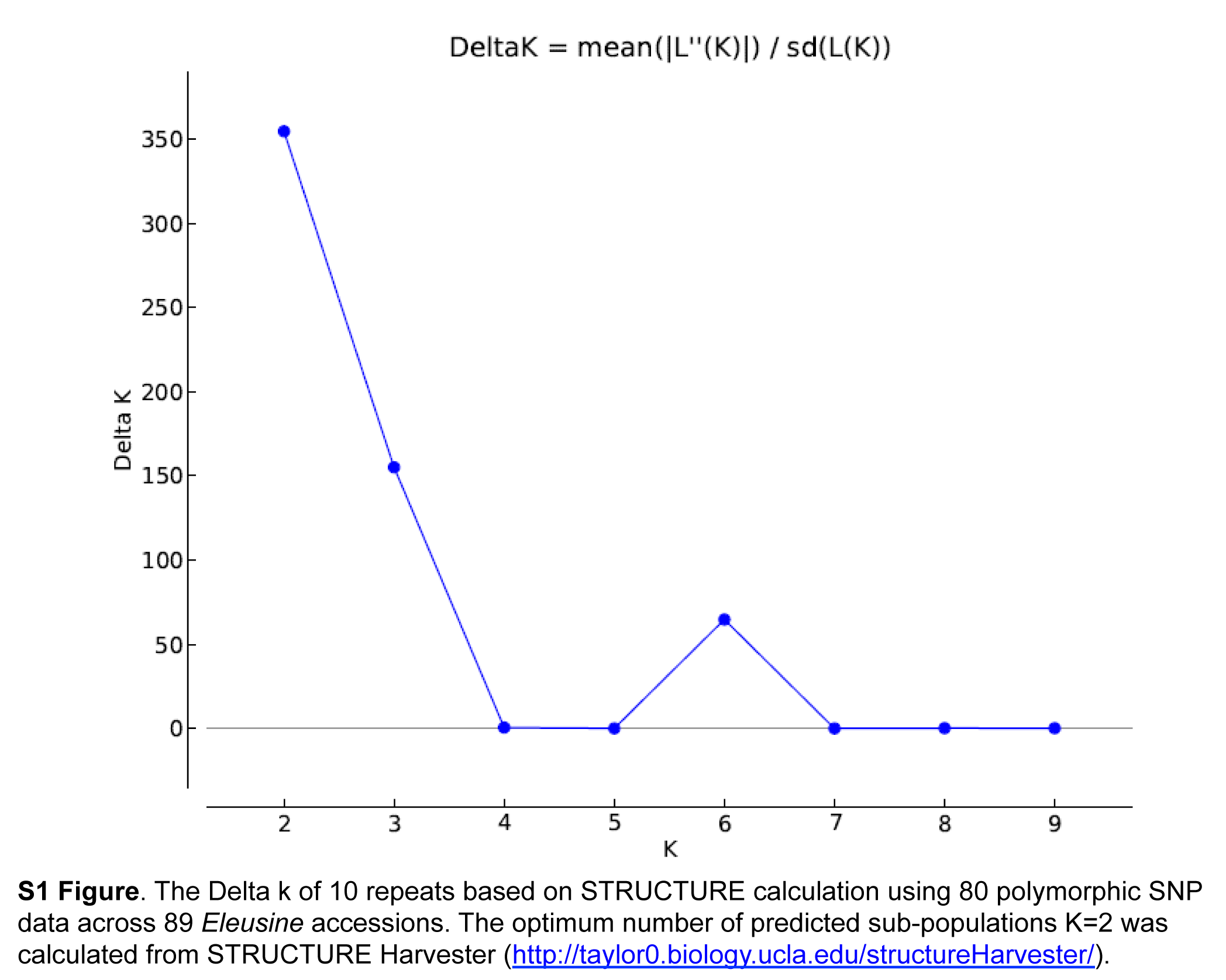

Supplement: S1 Fig — (TIF) [file pone.0159437.s001.tif]
