# Supplementary material for: Identification of SNP and SSR Markers in Finger Millet Using Next Generation Sequencing Technologies
Source: PLoS One. 2016 Jul 25;11(7):e0159437. doi: 10.1371/journal.pone.0159437 (PMC4959724; doi:10.1371/journal.pone.0159437)
Supplement: S3 Table — (DOCX) [file pone.0159437.s004.docx]

**S3 Table. A list of the 101 SSR primers supplied by Ecogenics and validated using 10 finger millet genotypes**

| **Name** | **Motif** | **Amplicon size** | **Forward Primer Sequence** | **Reverse Primer Sequence** |
| --- | --- | --- | --- | --- |
| ICECP1 | (TG)14 | 115 | TCGTGCCCTTTCTCTCTCTC | TGCAATCTGATCAACAGCCG |
| ICECP2 | (CA)22 | 104 | TTGACTTCCAGTACCCCGAG | GTTAGTGGAAAGGACCAAGCAG |
| ICECP3 | (CA)16 | 137 | GCACATTGTTCCTCTTCCCTC | TGGTACATCTCAACATGGTTTCTC |
| ICECP4 | (TC)11 | 119 | TCATGGCTGACCTCCCTTTC | TGCATCCCTTTTTGCTCTACTC |
| ICECP5 | (CA)14 | 123 | CTCCTCTGGTCACCTTCCTC | CGAGGGAGAAAATCCGTTGG |
| ICECP6 | (TC)13 | 120 | TTCTCTCCCACGTCGTTGTC | ATGTCGAAGGAGGAGCGTC |
| ICECP7 | (TG)13 | 124 | GGTCGTGAGCTTGTCCATTC | AGAGAACCCAGAGGGAGTAG |
| ICECP8 | (AG)12 | 217 | AAACCCACCCACCCATACTC | TCTATCTGCTGCCTGCTCTG |
| ICECP9 | (AC)11 | 177 | TTTGGATCAGCCGTTTCGC | AGGTCTCCACATTCTCCAGC |
| ICECP10 | (AC)11 | 87 | TGTAACAAGATGGCCGGAGG | ATCTACCGCGCAAGTACCAG |
| ICECP11 | (AG)19 | 219 | CCAGAACCAAATCCGTCACC | ACCGAGCCCACTCAGTTATG |
| ICECP12 | (GT)12 | 105 | TGCTTTACTGGCTCGATCTC | GGAGGAGGACGACACAAAAC |
| ICECP13 | (AG)14 | 145 | GACCTTCTACTTGTTGTCTCTCC | TATGGCATGGAGACACTGGG |
| ICECP14 | (GA)13 | 127 | AACCCGTGGCAGTGTTGC | AACCCTTCCCACTGTTCCTC |
| ICECP15 | (GT)15 | 111 | GCTCCAAGTCTCTAGTTGTTGG | TCTCATCTCTATGGGCGTTCG |
| ICECP16 | (CT)11 | 155 | GCCTTCTTCCTCACAGCATC | CAGGAGTAGAGCATGGACGG |
| ICECP17 | (AC)11 | 192 | CATATGTAATAGCCAAACCCCC | GGGTTCCCGTTACCCTTACC |
| ICECP18 | (AG)23 | 155 | GCGGATCATGTTGGGCATAAG | CTCGTGTCGTCGTCGTAGAG |
| ICECP19 | (AG)13 | 140 | TGCTTGTTTGGATCTTGCTC | CGACCACCACTCTCTCTCTC |
| ICECP20 | (GA)29 | 155 | TGCTTGTTTGGATCTTGCTC | GTAGAGACGACCGACCGAC |
| ICECP21 | (CA)17 | 102 | CATGTGCACCCATGTGAACG | TGGCTGTGTCTGTGAGTGC |
| ICECP22 | (AG)14 | 84 | GTTAATCGTGCTGGTCCTCG | ACCCCAACGATGTACTACCC |
| ICECP23 | (CA)14 | 115 | TACCTTACTTATCCCGCGCC | CTGGACCTGGACGGATCG |
| ICECP24 | (CT)12 | 240 | TCAATTTTGGGCTCGCCTTC | ACAGAAGATTGAACAGTGCGG |
| ICECP25 | (AC)13 | 115 | TTCGATACGGGGCGAAAATG | CTGCTGCTGCTGCTCATC |
| ICECP26 | (GAT)11 | 119 | AGCAAGAGCAAGAGAAACAAG | GCCAAGTCCTCAGTCAAGTC |
| ICECP27 | (AG)12 | 126 | GTGAGGTTTGGTTGCGAGTG | TCGACCTCCCGTCCTCTG |
| ICECP28 | (CA)12 | 98 | AGACGACTTCACGCAGCTC | GACACGAGCCGAAATGGAAC |
| ICECP29 | (AG)24 | 112 | TACTGCTCTTGTAGCAGGGG | TAGTACGACGACGACCGACC |
| ICECP30 | (AG)13 | 221 | ACAGTAGGAAATAAGTAATTGCATGAG | TGGCATGAACGTGTTGACAG |
| ICECP31 | (AC)11 | 131 | CAATTGCCCCAAACTTGAATTG | AGGTACCCTGTGAAGTCATCC |
| ICECP32 | (TG)11 | 88 | CCCAAGCGTCTCTCTCTCTC | TGTTCATACCTTACCAAAATAAATGG |
| ICECP33 | (AC)12 | 82 | ATCGGACATCCTTTTCTGGC | ACTGTGTGTGTGTTTGTCGTG |
| ICECP34 | (GTA)11 | 165 | ACACGGGAAAACGGGAAAAC | AGGAAGGAAGTATAGGGTAGGG |
| ICECP35 | (AG)26 | 134 | TAGAAAGACGGGAAGGAGCC | CGACCGACCACCACCTAC |
| ICECP36 | (GA)11 | 91 | GAAGTCCTCGTGTTGCATCC | CAAGCTCGGGCTCGTATAAC |
| ICECP37 | (TC)12 | 93 | CGACACGGTTTCCATTGGTC | GCAAAGAGGTAGCGAAGCTG |
| ICECP38 | (AC)20 | 142 | TGCATAGATTTGTGTTCTTTCTTGC | GGTCCGCATGTTAGTTACGTC |
| ICECP39 | (TG)11 | 96 | TCCGAGAAACTTTGTGATCGG | GACTCCCCCTATCCACGTC |
| ICECP40 | (AGG)8 | 129 | CAAGTCACCGTTGTCGCAG | ACTACTGCTTGCTCCTCGTC |
| ICECP41 | (AAG)8 | 131 | CAAGTCACCGTTGTCGCAG | ACACTACTGCTTGCTCCTCG |
| ICECP42 | (TG)13 | 86 | AGCTAGCCAGCCATTTGTCC | GCAAACGAACACAACAGTAACG |
| ICECP43 | (AG)12 | 80 | TCTTTTGCTTGTTTGGATCTTACTC | CTCGCTCTCTCTGTCTCTCTC |
| ICECP44 | (TG)13 | 83 | AGCTAGCCAGCCATTTGTCC | CAAACGACACAACAGTAACGC |
| ICECP45 | (AG)18 | 137 | TCGAATTATTGGCGCACAGG | TCTGTGAGACGACCACGAC |
| ICECP46 | (AC)12 | 121 | CCTCGTGTTGACGTGCAG | ACCACCGTGACCTTCCTTC |
| ICECP47 | (CA)21 | 84 | AATCACAGCAACCAGCAATC | TCGAGCTGTGTGTGAGTGAG |
| ICECP48 | (AC)11 | 117 | ACAGGAGCAGGTACAGATCG | TATCCAAACACAGCGTCAGC |
| ICECP49 | (GA)12 | 163 | AAACGCGAGAGAGTACAGGG | CTCGTGTCGTCGTCGTAGAG |
| ICECP50 | (AG)17 | 119 | CATCATTCTGCTTCCCCTGC | CGCGATGGATGATTGGATTG |
| ICECP51 | (AG)11 | 86 | TGCAAGCGTGGCTTCATTC | GGTCCGTCGTGTTCCTACTG |
| ICECP52 | (CA)21 | 93 | GGTGACCAGGATCATACCCC | GCTTTTACTTGAAGGCCCATTTG |
| ICECP53 | (CA)12 | 114 | TCTCAGTGGTATTTTGGTCCC | AGGTTGGTGGATCTGGATTTTG |
| ICECP54 | (AG)20 | 106 | CGCAAGCCGACAACAAAATG | AGAAGAAGCAGACACGACAG |
| ICECP55 | (AG)11 | 190 | CGTGTCGGGGGAGATAGAG | TTAAAACCCCCGGTAACCCC |
| ICECP56 | (TG)11 | 120 | ATCTCGTTGCATTCCGGTTG | TCAAGCCCTTATGCCCCC |
| ICECP57 | (CA)11 | 118 | TGATGGTGGTTGCCAGGTTC | CCAAGTGGTGTCGAGGAAAG |
| ICECP58 | (AC)20 | 105 | CGAATTCAGCTAGCGTGCC | GCTGAACCTTGTGCGGTG |
| ICECP59 | (CT)11 | 110 | GAGTCGCAATAGCTGAAGGC | TCAACGACCGGACGAAGAC |
| ICECP60 | (AC)12 | 82 | TAATTTGGGCTCGACCTTGC | CTTGTCGCGCGCATCATATC |
| ICECP61 | (GA)11 | 88 | CGCCGTGCTCACATCAGG | CCCTGCTTGTCAGTTCCTTC |
| ICECP62 | (TG)11 | 96 | ACAGACCTCCTCTCTCTCCC | ACCTGGATTGCTGATGGAGC |
| ICECP63 | (AG)13 | 119 | ACCAGATCCACCCACCATATC | ATCCGTCCCCTCTCTCCTAC |
| ICECP64 | (AC)17 | 96 | AGAAAACGGGAAAGATCCCAG | TTTCTCTGGCACCAGCAATC |
| ICECP65 | (GA)15 | 151 | ACACTAGAAAGAGAGAGAGAGAGTG | GTAGTAGACGACCGACCGAC |
| ICECP66 | (AC)13 | 100 | AGACAGCAGTTGTACCATCAC | CATGTTACGGAGAGGGGTCG |
| ICECP67 | (AG)12 | 160 | TGGAGAGAGAGTATCGTTTTGC | TACGTACGGAACGGAACGG |
| ICECP68 | (AC)15 | 83 | TGCATGTCATTAAAATGTATGTGTG | CTCAAAGCACCTCACAAGGC |
| ICECP69 | (CA)12 | 120 | TCACACGCACACCCACAC | AGGCTCATATGTAATCCTAACCTAC |
| ICECP70 | (TC)11 | 96 | TCAAGCTCGGGCTCGTATAG | AACTTGAAACCCGACGTTGC |
| ICECP71 | (AG)12 | 98 | CTGAACGAAGGCCTGTTTCC | TGTGTTGATGTTGGGTGTGC |
| ICECP72 | (TG)13 | 98 | TCCAATTCTGTCCCATCCCC | CGAACCACGTTGCTCACATC |
| ICECP73 | (AC)11 | 93 | TGTTTCTGTGAGCTATCTTTGGC | AAGACCCTGGTACGCATCTC |
| ICECP74 | (GA)11 | 191 | ATGCTCTTTCTCACGGAGCG | CGTGCGTTTGTCAAGTAGGG |
| ICECP75 | (CA)13 | 118 | CAGCATCCTCCAAAAGAGGC | GATCCATGTTAGCGTGCGTG |
| ICECP76 | (GT)15 | 250 | CCCATGACTACCGACAACCG | TAAATAGTCCGCCTCCCGTC |
| ICECP77 | (AC)14 | 104 | CCGTCTCCCAATCCTACCTG | GGTGGTGGTTTCAGGCTTTC |
| ICECP78 | (GT)22 | 112 | CCACCCTGAGTTGGATCTGG | TAACGTACCGACCGACCAC |
| ICECP79 | (CA)13 | 111 | CGGAACCAAGAGACAATCGC | ATACCCAAGCCACAACATGC |
| ICECP80 | (GA)13 | 97 | ACGAGAAAATGGAATCGCGG | CTTCTTGCTTGTGTCCTGCC |
| ICECP81 | (TG)18 | 128 | TTCTAGCAGTGTGTGTGTGC | AATGAAGCAGTGGGAGGGTC |
| ICECP82 | (GT)24 | 119 | AGGGGATGCTCCAAAGTCTC | GGAACCAGGAGACAATACGC |
| ICECP83 | (CT)13 | 149 | TCGCTCTCTCTCTCTGTGTG | ATTTGTCTGTCTCGTATCTTACTAC |
| ICECP84 | (TC)12 | 103 | TTCATGTGATGCAAGCCACC | CAGGCAAGATGCTGTTCTGG |
| ICECP85 | (TC)14 | 96 | CAGCAGCATCTATTTTCCATTGAC | GAAGAGAGGGAGCTTCGCC |
| ICECP86 | (TG)11 | 80 | TGTGAGTTCCTCTCTCTCTCTC | CCTAAGCAGGTTGCGTCTTG |
| ICECP87 | (AC)12 | 80 | ATCACTAAAGACCATAGCCAACC | CGTCATTCCTCTAGCGTGTG |
| ICECP88 | (GA)19 | 158 | GCTTCATGGGAGAAACTTGGG | CTTGCCGCCTCTCTCTCTG |
| ICECP89 | (GA)11 | 128 | CCACCAGAATCCAATGGCAC | TCGACTTTGTTTGCATGCTG |
| ICECP90 | (TG)17 | 220 | ATTCATCGACTCCCCAGTCC | AGCATGGACGAAGCGAAATC |
| ICECP91 | (GT)11 | 127 | AGATGAAAATGACTCGGTCTTGAG | CTTCTCAGTCCTTCACCCCC |
| ICECP92 | (AG)26 | 237 | CCCGTTTCCACCATCACAAC | GTACGACGACGACCGACC |
| ICECP93 | (AC)12 | 167 | AAGGAAGGAGAGGGCTCCAC | AGGGCCCACAGATAAACCTC |
| ICECP94 | (GT)12 | 128 | TGCTGGAGATCGCTGAAAAG | CGACCTTGCTTGCAAAAACC |
| ICECP95 | (GA)14 | 101 | GATGGCGGTTGTGATATACGG | GTCACCACTCCTCTCATCC |
| ICECP96 | (TC)12 | 134 | AGCAGGTCAGTAAGCTAGGC | CTCCGTGTGTCGTCGTAGAG |
| ICECP97 | (AG)12 | 122 | GTTACTTGGTAAACCGCCCC | AGTGTAGGAGTCAAAACAAAGC |
| ICECP98 | (AC)12 | 191 | CGAGTGAGTGTTCGTGTGTG | TGCATGAAATAGATGGGCCG |
| ICECP99 | (TC)11(TG)13 | 125 | ATCGACCTTTCCCTTCCTCC | TACTACAAGGGAGTTGGGCG |
| ICECP100 | (CT)14 | 83 | CACTCTCTCTCGCTCTCTCAC | CCGACCACGCACCGATTG |
| ICECP101 | (AAC)16 | 182 | AGGTTGTCGAACTGGAGACC | TAGCTGACCTATCGACGTGC |

**S4 Table.** **A list of 92 SNP markers from which finger millet KASP assays were developed**

| **SNP ID** | **Allele Y** | **Allele X** | **Sequence** |
| --- | --- | --- | --- |
| ICECSNT2 | C | T | AAGCCTAACAATGCA[T/C]GTGTAATGCTCCAT |
| ICECSNT3 | A | G | ATATTAGGTTGATGG[G/A]TTGGTAATTCTGAT |
| ICECSNT4 | C | A | TCCACATACATGCAG[A/C]AGCTGTTGACAACT |
| ICECSNT5 | T | C | TAACTTTTGTAGCCG[C/T]GCTTTGCCAATTAC |
| ICECSNT6 | A | G | ATCTTGATTTTTGGT[G/A]CAGCATATTCTTGC |
| ICECSNT8 | A | G | AAAAATAACAAATGT[G/A]TTTTTAGAAGGGTA |
| ICECSNT9 | G | A | TTTCATGACATATGA[A/G]CAGTATAGGTGCTG |
| ICECSNT11 | T | C | CATGGTTGATGCCAT[C/T]GATGAGTATGCTGT |
| ICECSNT12 | T | C | ATTTTTCTTTTGTAG[C/T]CCTATACTATATTT |
| ICECSNT13 | C | A | CATATCCAGTAGTTG[A/C]TTKAGATCTGCATA |
| ICECSNT14 | C | T | GAAACCTGAAGATGT[T/C]GCCACTATCTGCTA |
| ICECSNT15 | A | G | GATCTGATGCTGCTG[G/A]CACCGGGAAGATAG |
| ICECSNT16 | C | T | ACCACYAATTAACTA[T/C]AGTGKTCTGTATAT |
| ICECSNT17 | A | T | TTTAGTAATTTWRCC[T/A]ATGTTTCAAAATAA |
| ICECSNT18 | T | C | TAGCATAGCAAAACA[C/T]GRCATTTTACAATT |
| ICECSNT20 | C | T | TTGAATAACCCAATA[T/C]ATTCAGTARGGACC |
| ICECSNT22 | A | C | CCAGGTTTATAAGGT[C/A]ACACCTTGGAGTGA |
| ICECSNT23 | T | A | AACTTTTGCTAGCCC[A/T]TGTGAAATGGAATG |
| ICECSNT24 | G | A | ATAAACCTGTAATCC[A/G]TGTATCTTGAACGA |
| ICECSNT26 | A | G | CATTTTCTTCATGGC[G/A]AATGCAAGGCTCAG |
| ICECSNT27 | G | A | TCCGTCATGYTATTT[A/G]TTGCCYAATAACCC |
| ICECSNT28 | A | G | GGCTCCTTTTTACAG[G/A]TCGGGTTGCACACG |
| ICECSNT29 | A | G | TTCCAATTAATCAAT[G/A]TAACATGGGCAAKA |
| ICECSNT30 | T | A | GATTTTAAATATAGG[A/T]CCTCTAAGATATTT |
| ICECSNT31 | T | C | TCTTACCTTTTATGA[C/T]GCCCACAGCCCACT |
| ICECSNT32 | G | A | TTTATTGAGCCGAAA[A/G]TTACGTGTATTGAT |
| ICECSNT33 | A | G | TTTTCTATTGTTTCC[G/A]TGATATCACCAGGA |
| ICECSNT34 | G | A | ATGTTGATTTGATTC[A/G]GATGTATTTATTTA |
| ICECSNT35 | A | C | TACTCCAACTGCCGA[C/A]TTGTGCTTACATTT |
| ICECSNT36 | C | G | GATACCAATTAACTA[G/C]TGCTGTTCTTTTGC |
| ICECSNT38 | C | G | TCAGTCTCTTGTGAG[G/C]TTTCRTGCATAGAC |
| ICECSNT39 | A | G | TTACCATGTAGGCAT[G/A]CGTGCGACATTTTT |
| ICECSNT40 | G | A | AAATTGGAGTACCAG[A/G]TATCAAATTGCTGT |
| ICECSNT41 | T | C | CTATGCAAATCTACG[C/T]CAAATTGTCTGCAG |
| ICECSNT42 | C | G | ACCTTCCYAAGGAAA[G/C]ACAGGATAAGAGCG |
| ICECSNT43 | A | G | TGAGACCTGGGAGAG[G/A]GCTGACACCTATGC |
| ICECSNT44 | A | G | CTAGTGGCGGCGGCG[G/A]CGCCSGTGCCTCCA |
| ICECSNT45 | A | G | TTCTGAAGTCAAWAT[G/A]TCACTGTATTATTA |
| ICECSNT46 | A | G | GGGTTCAGTTTCAGA[G/A]GCTGCYRGACAGAA |
| ICECSNT47 | C | T | TATAGAGCGAACAAT[T/C]GARCATACAGAAAA |
| ICECSNT48 | T | C | AGTAAGAAAARCATA[C/T]TTATCATAACGAAT |
| ICECSNT49 | T | C | TRCAATTCAATATGT[C/T]GTCGATTTCTGTGT |
| ICECSNT51 | A | G | GTTYCTTTAAAGTAC[G/A]TTRTATTTGGGTTC |
| ICECSNT52 | T | C | GRCCATGCAATTTCC[C/T]GGRTTTTCACCAGT |
| ICECSNT53 | C | T | GGCTCAATGTGATTG[T/C]ATTGTTACTGATTT |
| ICECSNT54 | T | C | ACATGGGTCGAGGCA[C/T]GTAGATATCAYAAA |
| ICECSNT55 | A | G | TCTCCACCTGATCAC[G/A]CCACAATACACMTG |
| ICECSNT56 | C | G | GGATTGTTGCATAAC[G/C]AGGACACRGCTACC |
| ICECSNT57 | T | C | ACATGAACCATATTT[C/T]GAGAGGGTGACAAA |
| ICECSNT58 | T | A | TGAAACTTCAAGAGC[A/T]GAGGAAAGAAGAAS |
| ICECSNT59 | C | A | AAGAAACAGCCCYAT[A/C]TCTTCTTCGCAGGA |
| ICECSNT60 | G | C | ACAGATGCATAGAAA[C/G]CTGTARGCAGCTAC |
| ICECSNT61 | C | G | AGCCAGAAGAGATCA[G/C]AAAGGAGGAGTATG |
| ICECSNT62 | T | C | ATGCATTCATCTTTG[C/T]GTAATTGTTGTGGT |
| ICECSNT63 | C | G | AAAGTTGACCTTTGA[G/C]TATGAATGTCTAAT |
| ICECSNT64 | A | C | TCATACTTCATTCTA[C/A]CAAGGTTATGGTTA |
| ICECSNT66 | A | G | TTGTACCAAGCAGCC[G/A]TAGCKTGTAGTGRT |
| ICECSNT67 | G | C | TTAAGCTATGTTGTT[C/G]TTTTTGGTCAAATG |
| ICECSNT68 | A | G | ACTGAAAATAATGTT[G/A]AATWATATAGACAC |
| ICECSNT69 | C | A | CATAGGGTATCTTTC[A/C]CRAGTTTCCCTGTA |
| ICECSNT70 | C | A | GATGTAATCTGAAAT[A/C]GCCYTCTCATCCAC |
| ICECSNT71 | C | T | CGATTGATCAAATAA[T/C]GAATTGGCCCTCGG |
| ICECSNT72 | C | T | ATCCGTATACCAGGT[T/C]ATCCAGTCTTAACT |
| ICECSNT73 | A | C | GCTATACAATTACAA[C/A]ATGYCTTGGTTCCA |
| ICECSNT74 | G | T | GGWTCCAAGTCAAAA[T/G]CTAGCTCCATGAAA |
| ICECSNT76 | A | G | CATTGAAACCTTGCA[G/A]AGAGCCTTCACAAA |
| ICECSNT77 | T | G | TTCTTARCTATTACA[G/T]ATTTAAACTTAATT |
| ICECSNT78 | C | G | TGCAGAGTCCAAATT[G/C]TCAAAATTTGTTGA |
| ICECSNT79 | A | C | TATTTCTCTGCACAA[C/A]AGATTGACAAAATC |
| ICECSNT80 | C | T | GGGTGAGGAACGCAA[T/C]GGCAACCTTGTCGG |
| ICECSNT81 | C | A | CAAAACAAATAAGAC[A/C]TTGAAGGTTCCATA |
| ICECSNT82 | A | G | AAAACAGGGTACTGC[G/A]TCTGTTCGAGCGTT |
| ICECSNT83 | T | C | CTCACTTGATTTGTG[C/T]ACACAACAGGATCA |
| ICECSNT84 | C | T | CGGCACAACAATCTT[T/C]GGAAAGCCCATTTC |
| ICECSNT85 | A | C | TCTAAAGAACTTGAT[C/A]TTGTCCGTCATGAT |
| ICECSNT86 | C | A | CCCMGGAAATAAGCT[A/C]TATATGCTGATTTG |
| ICECSNT87 | C | T | GATGCAATTGAAGCA[T/C]TGAGGGTCTCRTAC |
| ICECSNT88 | G | A | GCTAACTCTYCCTAT[A/G]GTWCATCACTTTCT |
| ICECSNT89 | G | A | AGCCCTCCACATATC[A/G]ATAAAGAACACCTA |
| ICECSNT90 | T | C | RCCCCTTCTGCCCCA[C/T]TCTCCAAGGCCGGT |
| ICECSNT91 | C | G | AGCTGAGACAAACTT[G/C]GTAATCTCCAGCTT |
| ICECSNT92 | T | C | ATTGGACAACCCACG[C/T]AAGAACAAATTSRT |
| ICECSNT93 | C | G | TCGGTATCCCCTAAC[G/C]AGTCATTGAACAAG |
| ICECSNT94 | G | A | CACAAAAACCTCTGG[A/G]CTCCAGAGACTACA |
| ICECSNT95 | G | C | RTTTTATTTACTAGT[C/G]CYGAGTTTCAATAA |
| ICECSNT96 | C | A | ACTTAGATTACTTGA[A/C]ACACTGTTCTACTT |
| ICECSNT98 | C | T | GTATCCTCGTAGTTA[T/C]TGGCGCTTGATTGA |
| ICECSNT99 | C | G | CTGTTGTTAAACATA[G/C]AATYTGGAAATTGT |
| ICECSNT100 | G | A | GGAGAACCCAAAGGT[A/G]AAGTCCAATAGCTT |
| ICECSN98 | A | G | GGAGAGAAACGATTC[G/A]TGACATTGGGAAGA |
| ICECSN99 | C | A | AACATCAGCTTACTC[A/C]TTTTCTTGGGCCAG |
| ICECSN100 | C | T | CAAAAGCTTCCCAAT[T/C]TTGTCTACCAGCTC |
