# Supplementary material for: Identification of SNP and SSR Markers in Finger Millet Using Next Generation Sequencing Technologies
Source: PLoS One. 2016 Jul 25;11(7):e0159437. doi: 10.1371/journal.pone.0159437 (PMC4959724; doi:10.1371/journal.pone.0159437)
Supplement: S4 Table — (DOCX) [file pone.0159437.s005.docx]

**S4 Table.** **A list of 92 SNP markers from which finger millet KASP assays were developed**

| **SNP ID** | **Allele Y** | **Allele X** | **Sequence** |
| --- | --- | --- | --- |
| ICECSNT2 | C | T | AAGCCTAACAATGCA[T/C]GTGTAATGCTCCAT |
| ICECSNT3 | A | G | ATATTAGGTTGATGG[G/A]TTGGTAATTCTGAT |
| ICECSNT4 | C | A | TCCACATACATGCAG[A/C]AGCTGTTGACAACT |
| ICECSNT5 | T | C | TAACTTTTGTAGCCG[C/T]GCTTTGCCAATTAC |
| ICECSNT6 | A | G | ATCTTGATTTTTGGT[G/A]CAGCATATTCTTGC |
| ICECSNT8 | A | G | AAAAATAACAAATGT[G/A]TTTTTAGAAGGGTA |
| ICECSNT9 | G | A | TTTCATGACATATGA[A/G]CAGTATAGGTGCTG |
| ICECSNT11 | T | C | CATGGTTGATGCCAT[C/T]GATGAGTATGCTGT |
| ICECSNT12 | T | C | ATTTTTCTTTTGTAG[C/T]CCTATACTATATTT |
| ICECSNT13 | C | A | CATATCCAGTAGTTG[A/C]TTKAGATCTGCATA |
| ICECSNT14 | C | T | GAAACCTGAAGATGT[T/C]GCCACTATCTGCTA |
| ICECSNT15 | A | G | GATCTGATGCTGCTG[G/A]CACCGGGAAGATAG |
| ICECSNT16 | C | T | ACCACYAATTAACTA[T/C]AGTGKTCTGTATAT |
| ICECSNT17 | A | T | TTTAGTAATTTWRCC[T/A]ATGTTTCAAAATAA |
| ICECSNT18 | T | C | TAGCATAGCAAAACA[C/T]GRCATTTTACAATT |
| ICECSNT20 | C | T | TTGAATAACCCAATA[T/C]ATTCAGTARGGACC |
| ICECSNT22 | A | C | CCAGGTTTATAAGGT[C/A]ACACCTTGGAGTGA |
| ICECSNT23 | T | A | AACTTTTGCTAGCCC[A/T]TGTGAAATGGAATG |
| ICECSNT24 | G | A | ATAAACCTGTAATCC[A/G]TGTATCTTGAACGA |
| ICECSNT26 | A | G | CATTTTCTTCATGGC[G/A]AATGCAAGGCTCAG |
| ICECSNT27 | G | A | TCCGTCATGYTATTT[A/G]TTGCCYAATAACCC |
| ICECSNT28 | A | G | GGCTCCTTTTTACAG[G/A]TCGGGTTGCACACG |
| ICECSNT29 | A | G | TTCCAATTAATCAAT[G/A]TAACATGGGCAAKA |
| ICECSNT30 | T | A | GATTTTAAATATAGG[A/T]CCTCTAAGATATTT |
| ICECSNT31 | T | C | TCTTACCTTTTATGA[C/T]GCCCACAGCCCACT |
| ICECSNT32 | G | A | TTTATTGAGCCGAAA[A/G]TTACGTGTATTGAT |
| ICECSNT33 | A | G | TTTTCTATTGTTTCC[G/A]TGATATCACCAGGA |
| ICECSNT34 | G | A | ATGTTGATTTGATTC[A/G]GATGTATTTATTTA |
| ICECSNT35 | A | C | TACTCCAACTGCCGA[C/A]TTGTGCTTACATTT |
| ICECSNT36 | C | G | GATACCAATTAACTA[G/C]TGCTGTTCTTTTGC |
| ICECSNT38 | C | G | TCAGTCTCTTGTGAG[G/C]TTTCRTGCATAGAC |
| ICECSNT39 | A | G | TTACCATGTAGGCAT[G/A]CGTGCGACATTTTT |
| ICECSNT40 | G | A | AAATTGGAGTACCAG[A/G]TATCAAATTGCTGT |
| ICECSNT41 | T | C | CTATGCAAATCTACG[C/T]CAAATTGTCTGCAG |
| ICECSNT42 | C | G | ACCTTCCYAAGGAAA[G/C]ACAGGATAAGAGCG |
| ICECSNT43 | A | G | TGAGACCTGGGAGAG[G/A]GCTGACACCTATGC |
| ICECSNT44 | A | G | CTAGTGGCGGCGGCG[G/A]CGCCSGTGCCTCCA |
| ICECSNT45 | A | G | TTCTGAAGTCAAWAT[G/A]TCACTGTATTATTA |
| ICECSNT46 | A | G | GGGTTCAGTTTCAGA[G/A]GCTGCYRGACAGAA |
| ICECSNT47 | C | T | TATAGAGCGAACAAT[T/C]GARCATACAGAAAA |
| ICECSNT48 | T | C | AGTAAGAAAARCATA[C/T]TTATCATAACGAAT |
| ICECSNT49 | T | C | TRCAATTCAATATGT[C/T]GTCGATTTCTGTGT |
| ICECSNT51 | A | G | GTTYCTTTAAAGTAC[G/A]TTRTATTTGGGTTC |
| ICECSNT52 | T | C | GRCCATGCAATTTCC[C/T]GGRTTTTCACCAGT |
| ICECSNT53 | C | T | GGCTCAATGTGATTG[T/C]ATTGTTACTGATTT |
| ICECSNT54 | T | C | ACATGGGTCGAGGCA[C/T]GTAGATATCAYAAA |
| ICECSNT55 | A | G | TCTCCACCTGATCAC[G/A]CCACAATACACMTG |
| ICECSNT56 | C | G | GGATTGTTGCATAAC[G/C]AGGACACRGCTACC |
| ICECSNT57 | T | C | ACATGAACCATATTT[C/T]GAGAGGGTGACAAA |
| ICECSNT58 | T | A | TGAAACTTCAAGAGC[A/T]GAGGAAAGAAGAAS |
| ICECSNT59 | C | A | AAGAAACAGCCCYAT[A/C]TCTTCTTCGCAGGA |
| ICECSNT60 | G | C | ACAGATGCATAGAAA[C/G]CTGTARGCAGCTAC |
| ICECSNT61 | C | G | AGCCAGAAGAGATCA[G/C]AAAGGAGGAGTATG |
| ICECSNT62 | T | C | ATGCATTCATCTTTG[C/T]GTAATTGTTGTGGT |
| ICECSNT63 | C | G | AAAGTTGACCTTTGA[G/C]TATGAATGTCTAAT |
| ICECSNT64 | A | C | TCATACTTCATTCTA[C/A]CAAGGTTATGGTTA |
| ICECSNT66 | A | G | TTGTACCAAGCAGCC[G/A]TAGCKTGTAGTGRT |
| ICECSNT67 | G | C | TTAAGCTATGTTGTT[C/G]TTTTTGGTCAAATG |
| ICECSNT68 | A | G | ACTGAAAATAATGTT[G/A]AATWATATAGACAC |
| ICECSNT69 | C | A | CATAGGGTATCTTTC[A/C]CRAGTTTCCCTGTA |
| ICECSNT70 | C | A | GATGTAATCTGAAAT[A/C]GCCYTCTCATCCAC |
| ICECSNT71 | C | T | CGATTGATCAAATAA[T/C]GAATTGGCCCTCGG |
| ICECSNT72 | C | T | ATCCGTATACCAGGT[T/C]ATCCAGTCTTAACT |
| ICECSNT73 | A | C | GCTATACAATTACAA[C/A]ATGYCTTGGTTCCA |
| ICECSNT74 | G | T | GGWTCCAAGTCAAAA[T/G]CTAGCTCCATGAAA |
| ICECSNT76 | A | G | CATTGAAACCTTGCA[G/A]AGAGCCTTCACAAA |
| ICECSNT77 | T | G | TTCTTARCTATTACA[G/T]ATTTAAACTTAATT |
| ICECSNT78 | C | G | TGCAGAGTCCAAATT[G/C]TCAAAATTTGTTGA |
| ICECSNT79 | A | C | TATTTCTCTGCACAA[C/A]AGATTGACAAAATC |
| ICECSNT80 | C | T | GGGTGAGGAACGCAA[T/C]GGCAACCTTGTCGG |
| ICECSNT81 | C | A | CAAAACAAATAAGAC[A/C]TTGAAGGTTCCATA |
| ICECSNT82 | A | G | AAAACAGGGTACTGC[G/A]TCTGTTCGAGCGTT |
| ICECSNT83 | T | C | CTCACTTGATTTGTG[C/T]ACACAACAGGATCA |
| ICECSNT84 | C | T | CGGCACAACAATCTT[T/C]GGAAAGCCCATTTC |
| ICECSNT85 | A | C | TCTAAAGAACTTGAT[C/A]TTGTCCGTCATGAT |
| ICECSNT86 | C | A | CCCMGGAAATAAGCT[A/C]TATATGCTGATTTG |
| ICECSNT87 | C | T | GATGCAATTGAAGCA[T/C]TGAGGGTCTCRTAC |
| ICECSNT88 | G | A | GCTAACTCTYCCTAT[A/G]GTWCATCACTTTCT |
| ICECSNT89 | G | A | AGCCCTCCACATATC[A/G]ATAAAGAACACCTA |
| ICECSNT90 | T | C | RCCCCTTCTGCCCCA[C/T]TCTCCAAGGCCGGT |
| ICECSNT91 | C | G | AGCTGAGACAAACTT[G/C]GTAATCTCCAGCTT |
| ICECSNT92 | T | C | ATTGGACAACCCACG[C/T]AAGAACAAATTSRT |
| ICECSNT93 | C | G | TCGGTATCCCCTAAC[G/C]AGTCATTGAACAAG |
| ICECSNT94 | G | A | CACAAAAACCTCTGG[A/G]CTCCAGAGACTACA |
| ICECSNT95 | G | C | RTTTTATTTACTAGT[C/G]CYGAGTTTCAATAA |
| ICECSNT96 | C | A | ACTTAGATTACTTGA[A/C]ACACTGTTCTACTT |
| ICECSNT98 | C | T | GTATCCTCGTAGTTA[T/C]TGGCGCTTGATTGA |
| ICECSNT99 | C | G | CTGTTGTTAAACATA[G/C]AATYTGGAAATTGT |
| ICECSNT100 | G | A | GGAGAACCCAAAGGT[A/G]AAGTCCAATAGCTT |
| ICECSN98 | A | G | GGAGAGAAACGATTC[G/A]TGACATTGGGAAGA |
| ICECSN99 | C | A | AACATCAGCTTACTC[A/C]TTTTCTTGGGCCAG |
| ICECSN100 | C | T | CAAAAGCTTCCCAAT[T/C]TTGTCTACCAGCTC |
